# Innovative Strategies to Abolish Microbial Persistence in Biofilm Fortresses

**DOI:** 10.3390/biom16060887

**Published:** 2026-06-16

**Authors:** Diana-Antonia Costea, Valentina-Alexandra Badaluta, Ioana Zachia-Zlatea, Alina-Maria Holban, Lia-Mara Ditu, Veronica Lazar

**Affiliations:** 1 Faculty of Biology, University of Bucharest, Splaiul Independentei 91–95, R-050095 Bucharest, Romania; costea.diana_antonia@s.bio.unibuc.ro (D.-A.C.); badaluta.valentina@s.bio.unibuc.ro (V.-A.B.); zachia-zlatea.ioana22@s.bio.unibuc.ro (I.Z.-Z.); alina.m.holban@bio.unibuc.ro (A.-M.H.); veronica.lazar@bio.unibuc.ro (V.L.); 2 Doctoral School of Urban Planning, “Ion Mincu” University of Architecture and Urbanism, Academiei Street 18–20, R-010014 Bucharest, Romania

**Keywords:** biofilm, antimicrobial resistance evolution, prevention of biofilm development, novel anti-biofilm strategies, synergistic anti-biofilm approaches, combination therapies, natural remedies

## Abstract

Biofilms are structured communities of microorganisms embedded in a self-produced extracellular polymeric substance (EPS) matrix, whose development significantly enhances microbial resistance to antibiotics, disinfectants, and host immune defenses, posing major challenges in clinical, industrial, and environmental settings. Compared with planktonic cells, biofilm-associated microorganisms can exhibit up to 10- to 1000-fold increased tolerance to antimicrobial agents, contributing to the persistence of biofilm-associated infections (BAIs). These infections remain difficult to eradicate due to reduced penetration, altered metabolic states, and the presence of dormant or persister cells. Anti-biofilm strategies can be broadly classified into physical approaches (e.g., ultrasound, mechanical stress, and light-based approaches) that target biofilm structure; chemical and enzymatic methods (e.g., EPS-degrading enzymes) that destabilize the matrix; and biological and molecular strategies (e.g., quorum-sensing (QS) inhibitors, anti-virulence agents, bacteriophages, phage-derived antimicrobial molecules, antimicrobial peptides, and natural bioactive compounds) that modulate biofilm development and integrity by targeting regulatory pathways and matrix stability through distinct mechanisms of action. Natural compounds, including lactoferrin, lactoferrin-derived peptides, and probiotic and postbiotic fractions of lactic acid bacteria (LAB), as well as plant-derived metabolites, have shown promising anti-biofilm effects, with efficacy often enhanced through complementary or potentially synergistic interactions. However, despite these advancements, clinical translation remains limited. For example, BAIs account for approximately 80% of chronic infections, with high recurrence rates and therapeutic failure reported in device-associated infections and chronic wounds. These limitations highlight the need for clinically translatable, multimodal approaches that integrate structural biofilm disruption, antimicrobial targeting, and host response modulation to design more effective and sustainable anti-biofilm strategies.

## 1. Introduction

**Figure 1:**
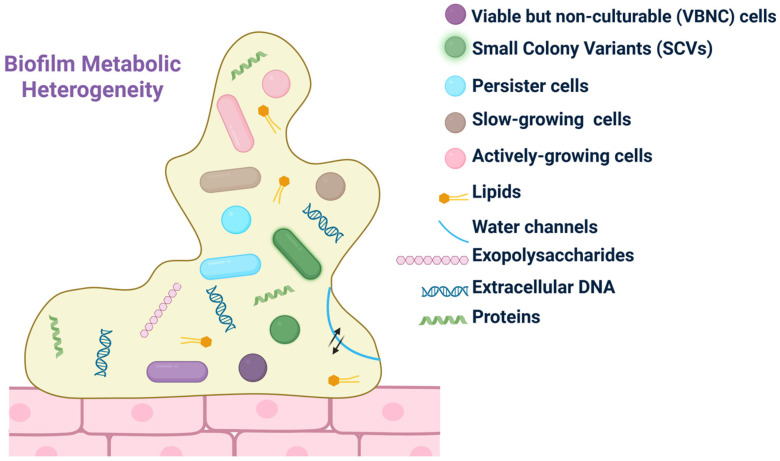
Metabolic heterogeneity in mature biofilms, illustrating slow-growing, persister, VBNC, and SCV bacterial subpopulations embedded in the EPS matrix. Figure generated using biorender.com.

Biofilms represent a dominant mode of microbial life and a major challenge across clinical, industrial, and environmental settings due to their complex architecture and pronounced physiological heterogeneity, which collectively confer exceptional tolerance to antimicrobial interventions [1,2,3]. At the molecular level, biofilm resilience is supported by EPS-mediated protection, QS-regulated community behavior, stress-adaptation responses, altered metabolic activity, genetic recombination and variation, and persister-cell formation [4,5,6]. Within mature, multilayered biofilms, nutrient and oxygen gradients generate physiologically heterogeneous subpopulations, ranging from metabolically active cells in superficial layers to slow-growing, dormant, or persister cells in deeper, anoxic, nutrient-depleted regions. Dormant biofilm-associated subpopulations comprise phenotypically distinct states, including persister cells, viable but non-culturable (VBNC) cells, and small colony variants (SCVs) (Figure 1). Although these populations share the ability to survive unfavorable conditions and antimicrobial exposure, they differ in their physiological characteristics, metabolic activity, culturability, mechanisms of persistence, and capacity for resuscitation. Persister cells are transient, phenotypic variants within bacterial populations that remain genetically antibiotic-susceptible but survive antimicrobial exposure by entering a dormant or slow-growing physiological state. Their tolerance is associated with metabolic inactivity, ATP depletion, activation of stress-response pathways, and growth arrest mechanisms. These cells are particularly abundant within biofilms, where nutrient limitation and environmental conditions further promote persistence. Following removal of environmental or antimicrobial stressors, persister cells can repopulate the biofilm, thereby contributing to infection persistence, therapeutic failure, and post-therapy relapse [7,8]. The VBNC state has been the subject of considerable discussion, particularly following the proposal by Song and Wood (2021) that some populations classified as VBNC may represent dead cells rather than viable dormant bacteria [9]. In response to this debate, recent efforts have sought to standardize the definition of the VBNC state. “Cells are characterized by their loss of culturability, ability to maintain a nonculturable state, and potential to resuscitate”, and are increasingly recognized as contributors to chronic and recurrent BAIs [10,11,12,13,14,15,16]. These criteria distinguish VBNC cells from dead cells and provide a framework for their identification. In addition, SCVs are slow-growing phenotypic variants characterized by atypical colony morphology, reduced metabolic activity, and increased tolerance to environmental stressors and antimicrobial agents. Some SCVs exhibit culture-dependent growth requirements, including elevated CO_2_ conditions or specific nutritional supplementation, which may hinder their detection under routine laboratory conditions. SCVs are strongly associated with biofilm formation and chronic infections, where prolonged stress exposure promotes metabolic adaptation and persistence. A well-characterized example is the CO_2_-dependent SCV phenotype of *Staphylococcus aureus*, which displays markedly impaired growth under standard culture conditions, but improved growth in CO_2_-enriched environments [10]. Such growth-dependent phenotypes are particularly relevant in biofilms, where metabolic adaptation to nutrient limitation, oxygen gradients, and environmental stress promotes the emergence of SCVs. Failure to recognize these growth requirements may lead to underestimation of SCV prevalence in chronic BAIs. Although SCVs share several features with persister cells, particularly enhanced stress tolerance and biofilm-associated survival, they are generally regarded as a distinct adaptive phenotype capable of reversibly switching to actively growing cells under favorable conditions. The relationship between SCVs and persister cells remains incompletely understood, with current evidence suggesting partial overlap between these survival strategies rather than complete equivalence [11,12,13,14]. Collectively, these dormant/persister populations are characterized by metabolic latency, reduced growth, or growth arrest, which decreases susceptibility to antimicrobials and contributes to biofilm tolerance and post-treatment regrowth [3,15].

These features undermine the efficacy of conventional therapies and contribute to chronic infection, relapse, and antimicrobial resistance emergence [16,17,18]. In the search for innovative strategies to address biofilm persistence, increasing attention has been directed toward the mechanisms that enable biofilms to withstand antimicrobial and host-derived stresses [4,5].

Conventional anti-biofilm strategies include chemotherapeutic agents and physical disruption methods. Antibiotics act mainly on conserved molecular targets essential for microbial viability and growth [19,20]. However, their efficacy is substantially reduced in BAIs, and even combination regimens frequently fail to eradicate mature biofilms [21,22]. In parallel, biofilms provide favorable conditions for horizontal gene transfer, thereby contributing to the dissemination of antimicrobial resistance [3,23]. Physical approaches, including mechanical stress, ultrasound, and light-based strategies, primarily destabilize biofilm structure and promote detachment rather than direct killing, and are therefore mainly explored as alternatives or adjuncts to antibiotics [4]. By contrast, novel and alternative anti-biofilm strategies aim to disrupt biofilm integrity, regulatory coordination, and persistence mechanisms. These include matrix-degrading enzymes, quorum-quenching (QQ) approaches, bacteriophages and phage-derived enzymes, and antimicrobial peptides, which act through diverse mechanisms, including matrix weakening, virulence attenuation, enhanced antimicrobial penetration, and selective killing of biofilm-associated cells [3,4,5,24,25,26,27,28,29,30]. Beyond the strategies discussed in detail in this review, additional approaches such as nanomaterial-based delivery systems, anti-adhesion or antimicrobial surfaces, immunomodulatory interventions, metabolic interference strategies, and CRISPR-Cas-based antimicrobials are increasingly explored within the expanding anti-biofilm landscape [6,31]. Recent research has increasingly focused on the individual or combined use of lactoferrin, probiotics, postbiotics, and/or natural bioactive compounds of plant origin for their anti-biofilm potential, as well as their supportive or adjuvant effects when combined with standard antimicrobial agents [32,33,34,35,36,37,38,39,40,41,42,43,44].

This review examines both conventional and emerging anti-biofilm strategies designed to interfere with biofilm resilience, including their effects on adhesion, biofilm integrity, and persistence when used alone or in combination, as well as their current limitations.

## 2. Conventional Anti-Biofilm Agents—Mechanism of Action and Current Limitations

**Figure 2:**
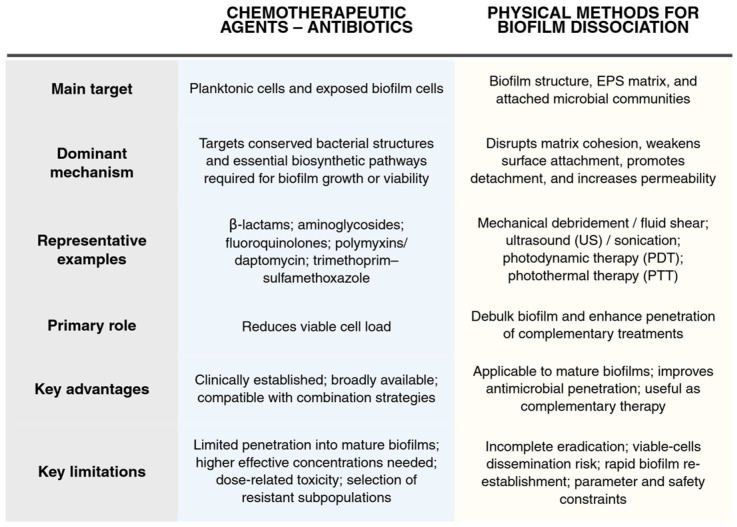
Conventional anti-biofilm strategies: comparative overview of chemotherapeutic agents [3,5,17,22,45,46,47,48] and physical methods [4,28,49,50,51,52,53] for biofilm dissociation, showing their main targets, dominant mechanisms, representative examples, primary roles, advantages, and limitations. Figure generated using biorender.com.

Conventional anti-biofilm strategies rely primarily on chemotherapeutic and physical interventions that target microbial viability or biofilm structure; however, their clinical effectiveness is frequently constrained by biofilm-specific tolerance mechanisms, limited penetration, and practical implementation challenges. The main conventional anti-biofilm strategies are compared using several criteria presented in Figure 2; however, both remain limited by biofilm-specific tolerance mechanisms and incomplete eradication when used alone.

### 2.1. Chemotherapeutic Agents—Antibiotics

Antibiotics remain central to antimicrobial therapy, but their efficacy in BAIs is limited by biofilm-specific tolerance mechanisms. Although chemotherapeutic agents are conventionally classified as bactericidal or bacteriostatic, their activity is more precisely understood through the essential bacterial processes they disrupt [19,20]. In planktonic cells, antibiotics target conserved pathways required for viability and growth, including cell wall synthesis, membrane integrity, protein synthesis, nucleic acid synthesis, and metabolism [19,20,45,46,47]. β-lactams exemplify cell-wall-active agents by binding penicillin-binding proteins (PBPs) and inhibiting the final transpeptidation/cross-linking steps of peptidoglycan synthesis, whereas polymyxins and daptomycin compromise membrane integrity [45,46]. Other antibiotic classes inhibit translation, interfere with DNA replication or transcription through targets such as DNA gyrase, topoisomerase IV, or RNA polymerase, or disrupt key metabolic pathways, as illustrated by trimethoprim–sulfamethoxazole-mediated inhibition of folate metabolism [45,46,47]. In biofilms, however, antibiotic activity is reduced by EPS-mediated penetration barriers, antibiotic sequestration or inactivation, nutrient–oxygen gradients, reduced metabolic activity, and dormant or persister cells [3,5,6,22,48].

Despite the broad spectrum of available antibiotics, clinical efficacy is predominantly assessed using planktonic susceptibility parameters, mainly minimum inhibitory concentration (MIC), which measures growth inhibition, and minimum bactericidal concentration (MBC), which measures bacterial killing under in vitro conditions. In biofilm contexts, complementary parameters such as minimum biofilm inhibitory concentration (MBIC), assessing inhibition of biofilm formation, and minimum biofilm eradication concentration (MBEC), assessing eradication of established biofilms, provide more biofilm-relevant information. However, these values may still underestimate in vivo therapeutic requirements [48]. For example, in orthopedic implant-associated *S. aureus* biofilms, in vivo MBEC values were substantially higher than in vitro MBEC values and increased with biofilm maturation, indicating that conventional susceptibility testing may underestimate therapeutic requirements in BAIs [54].

The frequently cited 10- to 1000-fold reduction in antibiotic susceptibility should therefore be interpreted as context-dependent rather than universal, varying according to bacterial species, strain, antibiotic class, biofilm maturity, and experimental conditions [3,16,17,55]. For example, a survey of 352 clinical *Pseudomonas aeruginosa* isolates showed highly variable biofilm-associated tolerance to ciprofloxacin, tobramycin, and colistin, ranging from MIC-level responses to values up to 16,000-fold higher than planktonic susceptibility estimates [56]. These observations explain why antibiotics considered effective against planktonic cells may fail to eradicate mature biofilms at clinically acceptable doses [5,22].

From an evolutionary and therapeutic perspective, these observations are clinically relevant because antibiotic exposure at MIC levels may allow survival of resistant subpopulations arising through spontaneous mutations, while increasing concentrations beyond the mutant prevention concentration is rarely feasible in vivo due to toxicity, tissue-damage risk, and ecological concerns related to antimicrobial residues [3,15,57]. To address these limitations, combination therapy and polytherapy have emerged as preferred strategies for managing BAIs, although even combination regimens may fail to eradicate mature biofilms when penetration, timing, or local concentrations are insufficient [21,22,58]. Specific examples of synergistic combinations and their underlying mechanisms are discussed in Section 4.4.

Beyond therapeutic failure, biofilms represent a critical ecological niche for horizontal gene transfer, facilitating the exchange of resistance and virulence determinants and contributing to the dissemination of antimicrobial resistance (AMR), including among ESKAPE pathogens [3,23]. Their tolerance to environmental stressors and broad-spectrum antimicrobials reinforces their role as persistent AMR reservoirs across clinical, agricultural, and environmental systems [5,55].

Taken together, these limitations underscore the need for enhanced surveillance, antimicrobial stewardship, and the development of innovative therapeutic approaches targeting biofilm formation, structure, and persistence beyond conventional antibiotic monotherapy [3,48].

### 2.2. Physical Methods for Biofilm Dissociation

Physical methods for biofilm dissociation disrupt biofilm integrity and promote detachment by targeting the mechanical and physicochemical properties of the biofilm matrix rather than directly killing microbial cells. These strategies, including mechanical stress, ultrasound, and light-based approaches, are explored as alternatives or adjuncts to antibiotics across medical, industrial, and environmental settings [4].

Mechanical stress-based strategies rely on the application of external physical forces (such as shear, compression, friction, and hydrodynamic pressure) to reduce the cohesive strength of the biofilm matrix. When the applied stress exceeds the biofilm’s mechanical failure threshold, detachment and dispersal of biofilm-associated cells can occur [49]. The effectiveness of these approaches is linked to the viscoelasticity of the biofilm, a property that allows it to deform, flow, or partially detach without complete eradication [49,50,51].

Mechanical clearance strategies are widely applied in both medical and industrial settings. For example, high-velocity fluid sprays used in dental hygiene or equipment cleaning generate intense shear forces capable of destabilizing biofilm structures within milliseconds. Such microsprays induce interfacial instabilities in biofilms, producing ripple-like structures in *Staphylococcus epidermidis* biofilms and wrinkle-like patterns in *P. aeruginosa* biofilms [51]. These microorganisms are frequently associated with medical device-related infections [50]. Rather than achieving complete removal, high-shear exposure may redistribute the biofilm across surfaces, enabling rapid mechanical recovery within minutes after stress removal [51].

In clinical practice, mechanical debridement (including surgical removal, gauze, lavage, ultrasound, as well as autolytic or chemical debridement) remains a widely used strategy for managing BAIs in chronic wounds. Although surgical debridement is considered the standard of care in wound management, it rarely eliminates biofilms entirely. Consistent with this limited standalone efficacy, debridement can temporarily reduce wound microbial burden, but biofilms may re-establish within 24–48 h, suggesting that residual biofilm often persists after physical removal attempts [51]. Combination approaches, such as debridement combined with antimicrobial dressings or skin grafts, can improve clinical outcomes, yet remain limited by persistent biofilm recurrence [51,58].

Ultrasound (US)-based approaches represent another class of physical anti-biofilm strategies with translational relevance in chronic wound management, dental biofilm control, medical-device decontamination, and biomaterial-associated infection contexts [4,52,53]. US therapy, or sonication, employs high-frequency sound waves that generate mechanical forces capable of disrupting biofilm architecture, primarily through cavitation phenomena affecting the EPS matrix [4]. Cavitation involves the formation, growth, oscillation, and implosive collapse of microbubbles within the liquid medium surrounding the biofilm, generating shock waves and high velocity microjets that exert intense mechanical stress [52,53]. In addition, elevated pressure and temperature generated during ultrasonic exposure further contribute to the erosion of biofilm components and the destabilization of the biofilm matrix [59]. These effects damage bacterial cells and the EPS matrix, while creating microchannels that enhance antimicrobial penetration [4,52,53].

The efficacy of US-mediated biofilm disruption depends on parameters such as frequency, intensity, pressure, exposure time, and distance from the target surface [53], as well as on medium-related factors, such as dissolved gas content, hydrostatic pressure, and liquid temperature [52]. Despite its potential, US therapy remains limited by several challenges. Standardized treatment protocols are lacking, and excessive exposure may cause unintended tissue damage (overheating, cartilage injury, or reduced implant longevity) [52,53]. Furthermore, induced fragmentation of biofilms can disseminate viable microbial cells, posing a risk of infection spread [53]. Recently, US has been investigated as an adjunctive strategy, as it can enhance the penetration and efficacy of antimicrobial agents [52,53]. In vivo, US combined with microbubbles enhanced vancomycin shows activity against device-associated *S. epidermidis* biofilms, reducing biofilm viability from 6.44 to 3.49 log10 CFU/catheter without evident histopathological damage, supporting US as an adjunct rather than a standalone anti-biofilm strategy [60].

Light-based anti-biofilm therapies have also gained increasing attention due to their non-invasive nature, spatiotemporal precision, and compatibility with multimodal therapeutic strategies [28]. Among these approaches, photodynamic therapy and photothermal therapy are the most extensively studied.

Photodynamic therapy (PDT) relies on a two-step process involving a non-toxic photosensitizer (PS), administered topically or locally, that generates reactive oxygen species (ROS) when activated by visible or near-infrared light at wavelengths depending on the absorption spectrum of the PS [61,62]. Generated ROS cause multitarget oxidative damage to polysaccharides within the EPS, lipids of cell membranes, proteins, and nucleic acids, ultimately leading to collapse of the biofilm matrix and/or microbial cell death [61].

Because this oxidative mechanism affects multiple cellular targets simultaneously, PDT exhibits broad-spectrum antimicrobial activity against bacteria, fungi, viruses, and protozoa, including drug-resistant strains, and can degrade virulence factors, disrupt phenotypic traits associated with multi-drug resistance (MDR), and interfere with metabolic processes essential for biofilm stability [61,63].

Clinically, PDT has been applied in the treatment of wounds, burns, oral, endodontic, and BAIs of medical devices [61]. However, its clinical use remains constrained by limited light penetration depth and by the risk of ROS-mediated phototoxicity in surrounding sensitive tissues, particularly when PS localization or light exposure is not precisely controlled, restricting PDT mainly to superficial, easily accessible, or device-associated biofilms [4,61].

Photothermal therapy (PTT) represents a complementary, non-invasive light-based strategy that relies on localized heat generation to achieve antimicrobial and anti-biofilm effects [62]. It uses photothermal agents or nanomaterials (PTAs) that convert absorbed light energy into heat and cause localized hyperthermia [28,64]. The mechanism of PTT is primarily mediated by hyperthermia-induced thermal ablation that denatures proteins, damages nucleic acids, and compromises membrane integrity, physically disrupting bioactive matrix components and enhancing permeability to antimicrobial agents [64,65]. This physical mode of action makes PTT less susceptible to resistance development, as it does not rely on specific molecular targets but instead physically damages bacterial cells [64].

A key advantage of PTT lies in the superior tissue penetration of NIR light compared with other wavelengths, enabling treatment of deeper or thicker biofilms, with reported penetration depths reaching the centimeter scale [64]. Nevertheless, successful application requires precise control of light intensity, exposure duration, and PTA concentration, as excessive heat may damage surrounding healthy host tissues [28,64].

**Figure 3:**
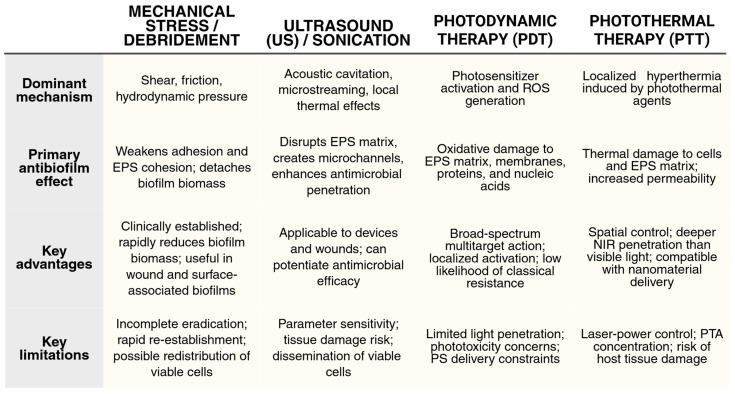
Physical anti-biofilm strategies: comparison of main mechanisms. Mechanical stress/debridement, ultrasound (US)/sonication, photodynamic therapy (PDT), and photothermal therapy (PTT) disrupt biofilm integrity through distinct physical mechanisms, including shear forces, acoustic cavitation, ROS generation, and localized hyperthermia [4,49,50,51,52,53,64]. Figure generated using biorender.com.

For both PDT and PTT, challenges related to PS and PTA delivery, biofilm penetration, and targeted activation continue to shape therapeutic use, and current research places them more as components of broader anti-biofilm interventions rather than standalone solutions. The characteristics and mechanisms of physical anti-biofilm strategies are presented in Figure 3.

## 3. Alternative Strategies Targeting Biofilm Integrity and Biofilm Persister Cells

**Figure 4:**
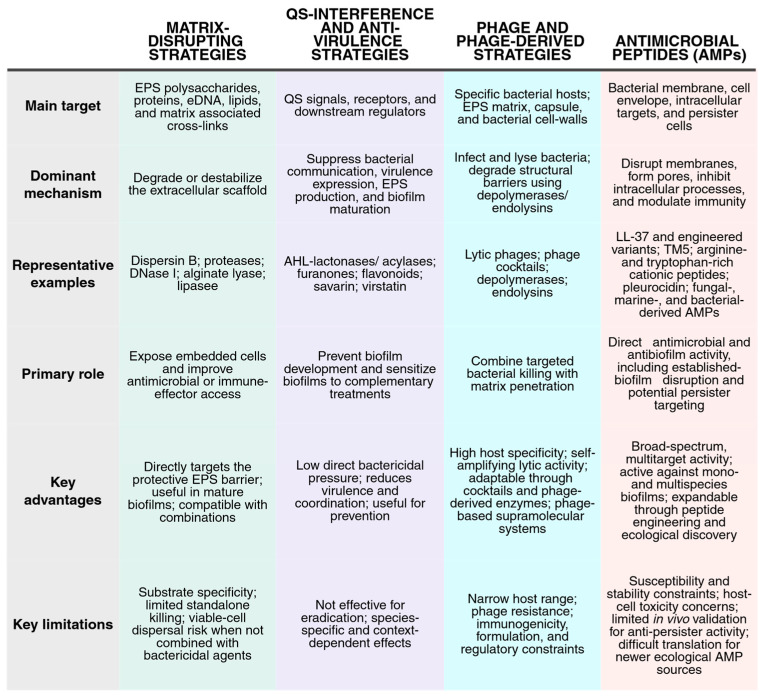
Alternative anti-biofilm strategies targeting biofilm integrity and persistence. Comparative overview of matrix-disrupting strategies, QS-interference and anti-virulence approaches, phage-based strategies, and antimicrobial peptides, summarizing their main targets, dominant mechanisms, representative examples, primary roles, advantages, and limitations [3,4,5,22,24,25,27,28,29,30,58,66,67,68,69]. Figure generated using biorender.com.

Novel and alternative strategies target biofilm integrity and persistence through mechanistically distinct but partially overlapping routes, emphasizing mechanistic intervention rather than direct bactericidal activity (Figure 4). For conceptual clarity, these approaches are grouped according to their dominant biofilm target and primary mode of action: extracellular matrix disruption, quorum-sensing-regulated communication, phage-mediated bacterial targeting, and membrane- or intracellular-targeting antimicrobial peptides and bacteriocins. Although several of the strategies discussed in this section exhibit multifunctional or overlapping effects, this organization provides a comparative roadmap for the section while acknowledging functional crosstalk and synergistic interactions between anti-biofilm strategies.

### 3.1. Matrix Degrading Enzymes

Matrix-degrading enzymes represent a major alternative anti-biofilm strategy because they directly compromise the extracellular scaffold that maintains biofilm cohesion. In natural biofilm communities, such enzymes are endogenously secreted by bacterial cells and retained within the matrix, where they contribute to matrix remodeling and biofilm life cycle regulation. By selectively degrading EPS components, these enzymes can trigger active biofilm dispersal, releasing cells that are able to recolonize new niches within the host or environment [1,25]. Therapeutically, this intrinsic process is exploited to weaken the physical and biochemical scaffold that protects embedded and persister cells, thereby improving their exposure to antimicrobial agents and host immune defenses [4,5]. By hydrolyzing EPS components such as polysaccharides, proteins, eDNA, and lipid-associated elements, these enzymes weaken intercellular adhesion, reduce mechanical stability, promote dispersal, and improve antimicrobial or immune effector penetration into deeper biofilm layers [4,66]. Because their primary action is matrix disruption rather than direct bacterial killing, they are best interpreted as complementary anti-biofilm strategies rather than standalone bactericidal agents [25,67].

Among the most extensively studied enzymes are polysaccharide-degrading enzymes, which hydrolyze matrix carbohydrates that play a central role in biofilm cohesion and architecture. These include glycoside hydrolases and polysaccharide lyases such as α-amylase, cellulase, dextranase, alginate lyase, hyaluronidase, and dispersin B. By cleaving glycosidic bonds within EPS polysaccharides, these enzymes reduce biofilm thickness, disrupt cell-to-cell aggregation, and promote detachment of mature biofilms across a broad range of clinically and industrially relevant microorganisms, including *Pseudomonas* spp., *Staphylococcus* spp., *Escherichia coli*, *Salmonella* spp., and *Listeria* spp. [3,66].

Dispersin B is a well-characterized glycoside hydrolase, originally identified in *Aggregatibacter actinomycetemcomitans*. It specifically hydrolyzes poly-N-acetylglucosamine (PNAG), a conserved matrix polysaccharide involved in intercellular adhesion and biofilm stability across diverse bacterial taxa [4,24,67,70]. Its exo- and endoglycosidic activities enable efficient PNAG cleavage during biofilm formation and dispersal. Experimental studies have demonstrated its ability to inhibit surface attachment, prevent biofilm maturation, detach preformed biofilms, and sensitize established biofilms to antibiotics, antiseptics, antimicrobial peptides, bacteriophages, and host immune mechanisms, highlighting its role in synergistic anti-biofilm approaches rather than as a standalone therapeutic agent [4,5,24,25,67]. Quantitatively, dispersin B showed strain-dependent activity, with 0.125–4 µg/mL detaching 24 h *S. aureus* SH1000 biofilms but not MRSA JE2 biofilms, while 1 µg/mL sensitized both strains to rifampicin–vancomycin killing [67]. Importantly, dispersin B has shown broad-spectrum activity against both Gram-positive and Gram-negative PNAG-producing organisms and has undergone extensive biocompatibility testing, supporting its potential for topical applications, particularly against staphylococcal biofilms [25,67].

Protein-degrading enzymes constitute another important group of matrix-targeting agents. Proteases such as proteinase K, subtilisin, trypsin, pepsin, pronase, aureolysin, lysostaphin, bromelain, papain, and serrapeptase hydrolyze structural proteins, which are fundamental components of the EPS matrix. They also degrade adhesion-associated proteins involved in surface attachment. This proteolytic activity leads to loss of biofilm structural integrity and reduced mechanical resilience. Multiple studies have demonstrated that proteases significantly reduce mono- and multispecies biofilms, including inter-kingdom bacterial–fungal biofilms, particularly when used in combination with other enzymes or antimicrobial agents [3,66]. Accordingly, proteases are considered among the most effective enzymatic approaches for biofilm disruption [70].

Proteinase K is a widely used reference serine protease, originally isolated from *Parengyodontium album* (formerly *Tritirachium album*). Its broad substrate specificity, including activity against native, denatured, and keratinous proteins, enables efficient degradation of proteinaceous components within the biofilm matrix [71]. In biofilm contexts, Proteinase K has been shown to inhibit early surface adhesion and microcolony formation, as well as to disperse 24–48 h established biofilms across several bacterial biofilm contexts [70]. Proteinase K at 2 µg/mL significantly inhibited biofilm development in *bap*-positive *S. aureus* V329 and several bovine mastitis isolates, but not in *bap*-mutant or weak biofilm-producing strains [72]. Although rarely applied as a standalone anti-biofilm agent, its proteolytic activity enhances biofilm permeability and facilitates deeper penetration of antibiotics, resulting in pronounced synergistic effects. Such combinations have been shown to promote effective degradation of preformed biofilms produced by a wide range of bacteria, including *S. aureus*, *E. coli*, *Staphylococcus lugdunensis*, *S. haemolyticus*, *Listeria monocytogenes*, *Gardnerella vaginalis*, and *Bdellovibrio bacteriovorus* [66,70].

Extracellular DNA (eDNA) is a critical structural component of many biofilm matrices, contributing to cohesion, surface attachment, and mechanical stability. Accordingly, nuclease-based approaches, including DNase I, micrococcal nuclease, restriction endonucleases, human DNase1L2, and prophage-encoded DNases, have been investigated for their ability to inhibit biofilm formation or detach preformed biofilms by degrading eDNA and increasing susceptibility to antimicrobial treatments [4,73]. Experimental studies have shown that DNase I alone can significantly inhibit biofilm formation by pathogens such as *P. aeruginosa*, *S. aureus*, *L. monocytogenes,* and *Campylobacter jejuni* [4,66], but its primary anti-biofilm value lies in matrix destabilization rather than direct bactericidal activity. DNase I exhibits pronounced synergistic effects when combined with antibiotics: disruption of the eDNA scaffold reduces matrix density and facilitates deeper antibiotic penetration, resulting in a 2- to 15-fold decrease in viable cell counts compared with antibiotic treatment alone [25]. Recombinant human DNase I inhibited *S. aureus* biofilm formation, with concentrations required for 90% reduction ranging from 0.125 to 4 µg/L, and detached preformed biofilms within 2–4 min at 1 mg/L [73]. DNase I has also demonstrated superior efficacy compared with protein- or polysaccharide-degrading enzymes in dispersing dual-species biofilms (such as *L. monocytogenes-E. coli* consortia), highlighting the central role of eDNA in complex biofilm architectures. However, enzymatic access to eDNA may be hindered by its association with polysaccharides and extracellular proteins, underscoring the rationale for combined enzymatic strategies targeting multiple EPS components simultaneously [66].

More recently, lipid-degrading enzymes have emerged as promising but less explored anti-biofilm agents. Lipases and esterases can disrupt lipid-mediated interactions within the biofilm matrix, contributing to biofilm destabilization across a wide range of pathogens. Lipases derived from environmental bacteria and fungi have demonstrated broad anti-biofilm activity and favorable stability profiles across diverse pH and temperature ranges, suggesting potential for both industrial and clinical applications [66].

In addition to enzymes that directly degrade matrix components, certain enzymes act primarily on bacterial cell walls and indirectly contribute to biofilm destabilization by weakening bacterial structural integrity and promoting secondary matrix collapse. Lysozyme, a host-derived innate immune enzyme, hydrolyzes peptidoglycan in Gram-positive bacteria, while phage- or bacterium-derived peptidoglycan hydrolases such as lysostaphin specifically cleave *S. aureus* cell walls, inducing bacterial lysis. Strong synergistic effects have been reported when cell wall–targeting enzymes are combined with antibiotics and chelating agents [4]. These observations indicate that enzymatic biofilm disruption is most effective when implemented within coordinated, multi-target strategies, an aspect further discussed in Section 5.

### 3.2. Quorum-Sensing Inhibitors and Anti-Virulence Agents

QS inhibition, often referred to as QQ, represents a major anti-biofilm and anti-virulence strategy that targets bacterial communication systems rather than bacterial viability. By interfering with QS-regulated coordination, this approach can reduce pathogenicity, biofilm maturation, and biofilm robustness without exerting direct bactericidal pressure [4,5,30].

Mechanistically, QS strategies are organized into three types of intervention: targeting QS signaling molecules, targeting QS signal receptors, and blocking downstream signaling cascades [26,28]. By disrupting signal synthesis, signal accumulation, signal perception, or downstream regulatory responses, QS inhibitors (QSIs) impair the transition from initial attachment to mature biofilm architecture and reduce the expression of QS-regulated matrix components, adhesins, and virulence factors. In contrast to matrix-degrading enzymes, QSIs do not physically dismantle the extracellular scaffold; instead, they indirectly weaken biofilm integrity by suppressing coordinated behaviors required for EPS production, stress tolerance, and community-level resilience, thereby sensitizing biofilms to antimicrobial agents and host immune clearance [3,4]. The latest proposed framework by Xu et al. is presented in Table 1; it provides an overview of the main QS signaling molecule classes and their known anti-QS and quorum-quenching (QQ) agents, covering both classical QS signaling molecules and specialized/emerging signals.

Targeting QS signaling molecules involves AHL-lactonases, oxidoreductases, and other molecular compounds that degrade or modify signaling molecules, thereby inactivating QS signaling through effects on autoinducers (AIs). Enzymatic QQ is particularly important at this level, and most studies focus on Gram-negative bacteria. In these systems, AHLs can be degraded by two types of hydrolases: AHL acylases and lactonases. AHL-lactonase, encoded by *aiiA* of *Bacillus* spp., effectively inhibits biofilm formation and attenuates virulence factors in several bacterial species, while AHL-acylase degrades AHLs by cleaving the amide bond, thereby reducing effective signal concentrations and modulating QS-dependent behavior [3,26]. By reducing effective AI concentrations below the activation threshold, these enzymes prevent QS-dependent modifications without directly affecting bacterial growth, a defining feature of enzymatic QQ strategies [28,30].

AHL-lactonase MomL, originally identified in marine bacteria, hydrolyzes the lactone ring of AHLs, autoinducers in Gram-negative bacteria, reducing biofilm formation and virulence without inhibiting bacterial growth [26,28]. As part of a strategy to modify existing QQ enzymes, engineered MomL variants (mutants MomLI144V and MomLV149A) have demonstrated enhanced efficiency and stability, and can block the production of virulence factors of *Pectobacterium carotovorum* [26].

QS receptor-targeting strategies act by inhibiting or competitively targeting signal receptors, thereby preventing downstream gene expression. Many QS receptors exhibit conserved ligand-binding domains, which enable pharmacological interference by structurally diverse compounds. Furanones and flavonoids are among the most studied QS inhibitors because they interact with QS receptors in a wide range of pathogenic bacteria [26,28].

Furanones, first isolated from the red alga *Delisea pulchra*, act as competitive inhibitors of LuxR-type receptors in Gram-negative bacteria, reducing QS signaling [22,28]. Synthetic halogenated furanones have been shown to impede bacterial swarming and QS-regulated behaviors by interrupting interactions between AHL molecules and regulatory proteins through competitive receptor binding [22], ultimately disrupting biofilm structure and promoting bacterial detachment [30]. Flavonoids, naturally occurring plant-derived QS inhibitors, can reduce biofilm biomass and persister populations in pathogenic biofilms like those formed by *S. aureus* and *L. monocytogenes* [95]. Drug-repurposing approaches have also identified compounds such as sitagliptin, which can interact with the LasR receptor and impair biofilm formation at sub-inhibitory concentrations [26,28].

Downstream QS interference targets response regulators or transcriptional control points after signal detection. Savarin, a small-molecule virulence inhibitor identified in *S. aureus*, suppresses QS by targeting AgrA, effectively disrupting *agr* operon-mediated signaling and inhibiting biofilm formation. Similarly, virstatin represses the expression of AnoR in *Acinetobacter nosocomialis*, attenuating QS-controlled signaling cascades and reducing biofilm formation and motility [26,28]. These features position QS inhibition as a modulatory anti-biofilm strategy, particularly relevant in integrated therapeutic frameworks discussed in Section 5.

### 3.3. Phage and Phage-Derived Antimicrobial Molecules

Bacteriophages (or phages) are bacteria-infecting viruses that have re-emerged as promising antimicrobial tools in response to escalating AMR [5,68]. First described in the early 20th century by Frederick Twort and Félix d’Hérelle, phages exploit the natural antagonism between bacteria and their viral predators, while their high host specificity enables selective targeting of pathogenic bacteria with limited disruption of the commensal microbiota [3,4,30,68].

Therapeutic applications predominantly rely on lytic phages, which infect bacterial cells and induce lysis, thereby reducing viable cells, contributing to biofilm architecture destabilization, and facilitating access to embedded bacteria [4,29]. In contrast, temperate phages are less suitable for therapy because their integration into the bacterial genome may facilitate horizontal transfer of virulence or antibiotic-resistance genes [3,29].

Phages exhibit anti-biofilm activity across multiple stages of biofilm formation, reflecting their ability to interact dynamically with biofilm structure and bacterial physiology. During early biofilm attachment and microcolony formation, naïve or weakly biofilm-embedded bacterial cells are easily infected and lysed through the action of endolysin and lysozyme-like (T4-like) enzymes, thereby inhibiting proliferation and preventing progression to mature biofilms [29]. As biofilms mature, penetration becomes increasingly challenging due to the dense EPS matrix. To overcome this barrier, many phages encode depolymerases, either as free enzymes or as tail-associated proteins, that degrade capsular polysaccharides, exopolysaccharides, and lipopolysaccharide components, facilitating phage diffusion into deeper biofilm layers [27,29,68]. This enzymatic degradation weakens biofilm cohesion, exposing shielded bacterial cells, and promotes further phage replication and biofilm disruption.

Phage-derived enzymes have attracted considerable interest as independent or adjunctive anti-biofilm agents. These include endolysins, peptidoglycan-degrading enzymes (including virion-associated peptidoglycan hydrolases (VAPGHs)), depolymerases, hyaluronidase, DNases, and lipases, which selectively degrade structural components of bacterial cells and biofilm matrices [22,27,29]. In addition to their direct lytic activity, subinhibitory concentrations of certain lysins have been shown to downregulate genes involved in biofilm formation, thereby impairing biofilm development [27]. Endolysins are particularly relevant because they can lyse bacterial cells, including metabolically dormant and persister cells, making them attractive for biofilm control [68]. By degrading protective matrix layers and weakening bacterial cell walls, these enzymes can facilitate phage penetration, amplifying bactericidal activity [29]. These properties have prompted the exploration of phage-derived enzymes as antimicrobial coatings for medical devices and bioactive surfaces, as well as disinfectants in healthcare and food-industry settings [27].

Recently, phage-based supramolecular systems (PBS), which combine bacteriophages with antimicrobial peptides, nanomaterials, or other functional enhancers, have been proposed to broaden anti-biofilm activity and improve biofilm penetration [29]. In parallel, nanoparticle-assisted phage delivery is increasingly explored to improve phage stability, targeting, biofilm penetration, and therapeutic efficacy [31]. However, their clinical translation remains constrained by immunogenicity and potential toxicity concerns [29]. In later stages of the biofilm life cycle, such as biofilm dispersal, phage cocktail therapies (comprising multiple lytic phages with complementary host ranges and enzymatic capabilities) have demonstrated superior efficacy compared with monophage treatments. Such cocktails broaden the host range, reduce the likelihood of phage resistance, and enhance biofilm eradication through combined enzymatic and lytic attacks [29,68]. Experimental studies have shown that a cocktail containing a depolymerase-producing phage (Pa29, which targets *P. aeruginosa*) and a non-depolymerase-producing phage (KP01K2, which targets *Klebsiella pneumoniae*) can effectively disrupt polymicrobial biofilms formed by both pathogens [29]. Accordingly, phage-based approaches are best understood as adaptable anti-biofilm tools whose broader therapeutic integration is considered in Section 5.

### 3.4. Antimicrobial Peptides

Antimicrobial peptides (AMPs), also known as host defense peptides, represent a diverse and evolutionarily conserved class of innate defense molecules with broad-spectrum antimicrobial and anti-biofilm activity. Historically, the discovery of lysozyme by Fleming in 1929 (an antimicrobial enzyme rather than a canonical AMP) [96], and the subsequent identification of gramicidin by Dubos and Hotchkiss in 1939 marked the early recognition of naturally occurring antimicrobial peptides with selective antimicrobial activity [69,97]. Produced by virtually all forms of life, AMPs function as rapid-response effectors of host immunity and have gained renewed interest as alternative or adjunctive agents against BAIs, particularly in the context of rising AMR [3,5,24].

AMPs exert anti-biofilm activity through multiple mechanisms, depending on the specific antimicrobial agents used: they can disrupt surface attachment, alter outer membrane proteins, downregulate QS molecules by inhibiting signal molecules, disrupt the transport of biofilm precursors into the extracellular environment, and enhance the host immune response [4,5]. Most AMPs are cationic and amphipathic, enabling electrostatic interaction with negatively charged bacterial membranes, followed by membrane permeabilization, pore formation, or complete membrane disintegration. Several AMPs translocate into bacterial cells, where they interfere with DNA, RNA, protein synthesis, or metabolic pathways [22,58,69]. In addition to their antimicrobial activity, these compounds also exhibit immunostimulatory properties, including acting as chemoattractants and activating classical complement pathways, as well as anti-inflammatory effects [3,69]. AMPs are structurally diverse and are commonly grouped into α-helical, β-sheet, extended, and cyclic peptides [4]. Numerous natural and synthetic AMPs have demonstrated anti-biofilm activity against clinically relevant pathogens such as *S. aureus*, *P. aeruginosa*, *E. coli*, *Acinetobacter baumannii*, and *Candida* spp., affecting both mono- and multispecies biofilms [58].

Human cathelicidin LL-37 is the only human cathelicidin antimicrobial peptide and a key effector of the innate immune system, produced mainly by neutrophils and epithelial cells and activated from the hCAP-18 precursor at infection sites. LL-37 exerts direct antimicrobial activity primarily through membrane disruption, while separately modulating host immune responses [98,99]. It displays anti-biofilm activity across diverse microorganisms, with *S. aureus* and *P. aeruginosa* representing well-studied examples, and can disrupt established biofilms while showing synergy with antibiotics, nanoparticles, and bacteriocins [98]. Engineered LL-37 variants with improved resistance to *P. aeruginosa* proteases further illustrate how peptide optimization can improve stability and therapeutic performance while reducing host-cell toxicity concerns [31].

Beyond classic examples, recent studies highlight unconventional ecological reservoirs of AMPs with anti-biofilm potential. Freshwater lignicolous fungi such as *Longipedicellata megafusiformis* and *Wicklowia fusiformispora* have been identified as sources of structurally novel peptides [100]. Marine-derived AMPs further illustrate the value of underexplored ecological reservoirs, as they may exhibit enhanced stability under harsh environmental conditions and combine membrane disruption, anti-biofilm activity, QS interference, immune modulation, and potential activity against persister cells, although their clinical translation remains limited by production scalability and validation gaps [101,102]. For example, pleurocidin, a winter flounder-derived AMP, reduced *Streptococcus mutans* biofilm by 75.2% at 64 µg/mL and showed anti-biofilm activity against drug-resistant *S. aureus* through mechanisms involving membrane disruption, metabolic interference, and QS modulation [102,103]. In parallel, endogenous AMPs produced by bacteria such as *Bacillus cereus* illustrate the role of antimicrobial peptides in inter-microbial competition and biofilm control [104]. Collectively, these findings expand the AMP discovery landscape and point toward next-generation anti-biofilm agents derived from underexplored ecological niches.

Cationic AMPs are among the few anti-biofilm agents shown to directly target persister cells, thus acting as anti-persister agents due to their electrostatic interactions with oppositely charged components of the cell membrane and wall components, leading to membrane pore formation. This mechanism enables AMPs to kill metabolically dormant persister populations residing in the deeper layers of biofilms. Broad-spectrum peptide TM5, as well as arginine- and tryptophan-rich cationic membrane-penetrating peptides, have demonstrated efficacy against persister cells in biofilms formed by both Gram-positive and Gram-negative bacteria, although in vivo validation remains limited [28]. These properties place antimicrobial peptides at the interface of direct antimicrobial action, anti-virulence activity, and immune modulation, with broader translational considerations discussed in Section 5.

## 4. Natural Bioactive Compounds—Emerging Agents in Modulating Biofilm Formation

As MDR, a major aspect of AMR, imposes a public health concern, there is an urgent need to discover and implement novel antibiotic alternatives [33,105,106,107]. AMR has been briefly defined as a “silent pandemic”, being considered one of the top 10 threats to public health [108,109,110,111]. According to the World Health Organization (WHO), the emergence of MDR microorganisms might lead to a “post-antibiotic era” in which current antibiotic agents would no longer be effective [108,112]. The spread of MDR bacteria is a result of the selective pressure exerted by the therapeutic use, either targeted or empirical, of broad-spectrum antibiotics [110]. In this context, natural bioactive compounds, such as lactoferrin, probiotics, postbiotics, and plant-derived compounds, might be promising alternatives or adjuvants to the standardized antibiotic therapy [33]. The significant non-genetic tolerance of biofilm-embedded cells to antimicrobials and immune defenses, which leads to chronic and recurrent infections, is a further reason to use natural bioactive compounds [3,33,105,113,114].

### 4.1. Lactoferrin

**Figure 5:**
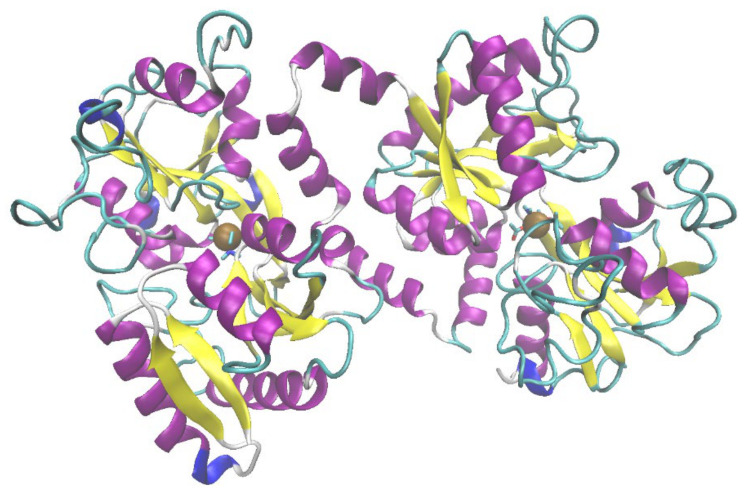
Biochemical 3D architecture of iron-bound bovine lactoferrin (holo-bLf) [123]. https://www.rcsb.org/structure/1BLF (accessed on 16 January 2026). Structures visualized with VMD 2.0 [124].

Lactoferrin (Lf) is a glycoprotein with a molecular weight of 80 kDa and iron-binding properties, identified for the first time in bovine milk, as bovine Lf (bLf), by Sorensen and Sorensen 87 years ago [105,115,116]. Lf has also been identified in the milk of other mammals, alongside several other secretions, where it ensures a first line of anti-pathogenic defense [117,118,119]. The polypeptide chain of Lf contains two globular lobes. Each lobe reversibly binds one ferric (Fe^3+^) ion and comprises two domains (N1, N2, and C1, C2) [105,117,120,121] (Figure 5). Depending on its Fe^3+^-binding state, Lf exists in two forms: (i) iron-free (apo-Lf), with a more flexible conformation and more susceptible to denaturation, and (ii) bound to two Fe^3+^ ions (holo-Lf), with a more stable structure, the bound ions being harder to dissociate [119,120,122].

Past and current research has focused on the multifaceted functions of Lf, as it exerts antipathogenic, anti-inflammatory, antioxidant, antineoplastic, and immunomodulatory activities [105,125,126,127,128]. Lf can maintain the homeostasis of iron, but also other microelements (Cu^2+^, Zn^2+^, Mn^2+^, Al^3+^, Ce^4+^), thus having a 300 times greater Fe^3+^ binding ability at a lower pH than transferrin [117,120,122,128]. Lf is released at infection sites via the secondary granules of neutrophils, being stored at 3–15 µg/106 neutrophils [105,129].

The antimicrobial action of Lf includes both bactericidal and bacteriostatic activities [126,130,131,132]. The amino end of Lf provides the molecular positive charge and ensures the bactericidal function because of its interaction with negatively charged cell wall components of Gram-negative bacteria (LPS, lipopolysaccharides) and Gram-positive bacteria (LTA, lipoteichoic acids) [127,130,131,132,133,134]. When Lf binds LPS in Gram-negative bacteria, it determines their release, disrupting the cell surface and causing high membrane permeability, thereby exposing the cell to osmotic shock or the bactericidal activity of antibiotics [120,135]. In Gram-positive bacteria, the interaction of Lf with LTAs induces the loss of surface charge and membrane integrity, allowing the enzymatic degradation of the peptidoglycan under the action of lysozyme [122]. Membrane porins play the role of Lf receptors, as the interaction with their receptors is associated with antibacterial activity of this glycoprotein [127]. The existence of two hLf and bLf binding sites on the LPS in the *E. coli* 055B5 cell wall has also been described [136]. The iron-chelating function of Lf is connected to the bacteriostatic activity, though the inhibition is transient as bacterial viability can be reinstated by external iron supplementation [130,131,132,134,135]. Bacteria are capable of scavenging iron for several vital mechanisms, including nucleic acid and ATP synthesis and O_2_ transport, through extracellular secretion of siderophores [105,121].

**Figure 6:**
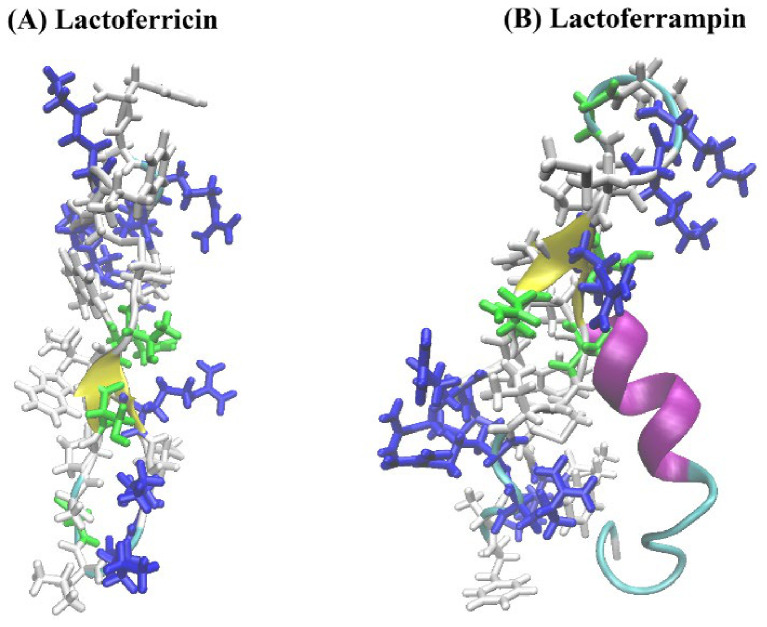
Biochemical 3D architecture of lactoferrin-derived peptides: (**A**) LFcinB, https://www.rcsb.org/structure/1LFC (accessed on 16 January 2026) [140]; (**B**) LFampinB, https://www.rcsb.org/structure/2MD1 (accessed on 16 January 2026) [141]. Structures visualized with VMD 2.0 [124].

Lactoferricin (LFcin) and lactoferrampin (LFampin) are antimicrobial peptides derived from the amino terminus of Lf through pepsin digestion [137,138]. These small peptides possess an α-helical conformation, are amphiphilic molecules, and, similar to the native protein, have a cationic charge, which explains their antimicrobial activities [120,138]. LFcin is a 25-amino acid-long peptide (residues 17–41), made out of primarily basic amino acids such as arginine and lysine, but also hydrophobic amino acids including phenylalanine and tryptophan [120,121,138,139]. In the LFcin structure, a disulfide bridge stabilizing a loop region is present, together with an amphipathic α-helix [120,138,139]. In contrast to the intact protein, LFcin lacks the iron-chelating function [120]. LFampin is spatially close to LFcin and has been isolated from the Lf N1 domain (amino acids 268–284), although a longer, more biologically active peptide (residues 265–284) with an N-terminal α-helix has been described. The helical fragment in their structure has been related to the inhibitory action of LFcin and LFampin against Gram-positive pathogens [138] (Figure 6).

The inhibitory effects of LFcin on bacterial growth have been extensively studied and demonstrated on several opportunistic pathogens, including *S. aureus*, *Enterococcus faecium*, *P. aeruginosa,* and *E. coli* [137,142,143,144]. In fact, one study aimed to evaluate the efficacy of the bLf-derived AMP (LFcinB), LFcinB-derived synthetic peptides, and the native form of bLf against *E. coli* growth, pointing to a dose-dependent effect of the native glycoprotein. The short synthetic peptides had a biological activity at the lowest dose (MIC = 6.2–25 mg/mL), followed by LFcinB (MIC = 100 mg/mL) and bLf (MIC > 200 mg/mL), reinforcing the bactericidal function of this Lf-derived AMP [137].

**Figure 7:**
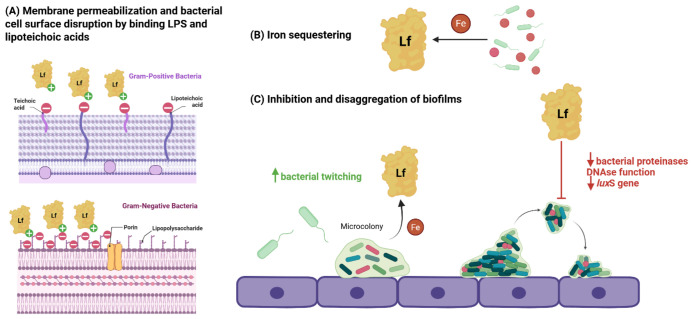
The antimicrobial and anti-biofilm effects of Lf are described above. Figure generated using biorender.com. Adapted with modifications from [33,120,129].

Biofilm formation on viable or non-viable substrates is a determinant of chronic and persistent infections; thus, the anti-adhesion properties of Lf and Lf-derived AMPs have been of particular interest for research since the past century [33,130,143,145,146,147]. The presence of iron is crucial for biofilm formation and viability; thus, Lf can reduce the survival of biofilms by sequestering iron [121]. For example, in *P. aeruginosa*, Lf prevented the formation of biofilms by triggering a motility behavior described as ‘twitching’, mainly related to its iron-binding property. In the absence of iron, the normal attachment of bacteria, followed by the formation of microcolonies and subsequent biofilms were inhibited [148]. Additionally, independent of its iron-sequestering ability, several species-specific anti-biofilm mechanisms of Lf have been described in previous in vitro studies. In a bacterial model of *Porphyromonas gingivalis*, the anti-proteinase activity of bLf was responsible for its biofilm inhibitory effect, contrary to its previously documented susceptibility to proteolysis [147]. In another report, Lf has been portrayed as a potential prophylactic agent against pneumococcal infections generated by persistent biofilms; the matrix of biofilms generated by *Streptococcus pneumoniae* possesses eDNA, which allows the acquisition of antibiotic resistance genes. The work of Angulo-Zamudio et al. shed light on the DNase activity of Lf, alongside its ability to prevent adhesion. At physiological concentrations, bLf eradicated *S. pneumoniae* biofilms established on inert and cellular substrates, as well as biofilms formed by MDR *S. pneumoniae* strains. The DNase function was demonstrated by the absence of eDNA in the biofilms of the test group compared with the untreated control [130]. In addition, in *S. pneumoniae*, it has been presumed that the disruption of biofilms might be related to bLf inhibiting the gene expression of *luxS*, a gene encoding a virulence factor modulating biofilm formation [149]. The abovementioned anti-biofilm mechanisms of Lf are summarized in Figure 7.

Lf has been proven to be effective in inhibiting the adherence and growth of several bacterial strains, including *E. coli*, *P. aeruginosa*, *S. aureus*, *E. faecium*, *Vibrio parahaemolyticus*, *B. subtilis*, *Streptococcus agalactiae*, *P. gingivalis*, *Prevotella intermedia*, *Helicobacter pylori*, *Salmonella enterica,* and *K. pneumoniae* [32,117,130,146,147,150,151,152,153,154,155]. The effects of Lf and Lf AMPs on the adhesion of conditionally pathogenic bacteria have been evaluated in multiple studies with heterogeneous methodology (e.g., MBEC, percentage inhibition, biomass reduction); these effects are described in Table 2.

Several studies shed light on the ability of Lf to inhibit pathogen adhesion to host cells, as it is the first step toward initiating an infection [161]. An early study emphasized the restraining effect of bLf at 16 mg/mL on enterotoxigenic and enteropathogenic *E. coli* adhesion to JTC-17 cervical cancer cells [162]. It has also been demonstrated that bLf mitigates the invasiveness of *P. aeruginosa* in A549 lung carcinoma cells [161]. In our recent paper, we demonstrated a 50% reduction in *P. aeruginosa* and *E. coli* adhesion to HCT-8 colon cancer cells when treated with Lf [33].

### 4.2. Probiotics and Postbiotics

#### 4.2.1. Probiotics—Characterization and Mechanism of Action

E. Metchnikoff (1907) described the concept of beneficial microorganisms for the first time, while the term “probiotic”, meaning “for life” in Greek, was introduced later (1953) to describe live microorganisms essential for the proper evolution of biological systems [163,164,165,166]. Currently, the FAO (Food and Agriculture Organization)/WHO definition of probiotics is widely accepted: “live microorganisms which, when administered in adequate amounts, confer a health benefit on the host” [163]. Probiotics are microaerophilic, non-sporulating bacteria, being part of the normal gut microbiota [163,167,168]. Probiotics benefit the host via several activities, including modulating immunity and inflammation, as well as inhibiting pathogen adhesion and mutagenesis under certain conditions [163,169]. Fermented foods, mainly dairy products, pickled products, and meats, are the main source of probiotics. The most well-characterized and used probiotic genera for human therapeutics include Gram-positive *Lactobacillus*, *Streptococcus*, *Enterococcus*, *Pediococcus*, *Lactococcus*, and *Bifidobacterium*, as well as the Gram-negative *E. coli* Nissle 1917 strain, and *Saccharomyces boulardii* strains [168,170,171,172].

*Lactobacillus* spp. and *Bifidobacterium* spp. are important constituents of the developing gut microbiota and are widely used probiotic genera [170,171,173]. *Lactobacillus* is the typical representative of Lactic Acid Bacteria (LAB) and is a genus belonging to the *Lactobacillaceae* family of the Firmicutes phylum [163,170,171]. Several metabolic, competitive, and antimicrobial mechanisms by which *Lactobacillus* spp. inhibit pathogenic microorganisms have been described. For instance, *Lactobacillus* species can inhibit pathogen attachment and colonization through the production of lactic acid from the metabolism of hexose carbohydrates, generating acidic conditions [33,36,170]. Additionally, lactobacilli produce hydrogen peroxide and benzoic acid, blocking the adherence of pathogens that are catalase-negative and acid-sensitive. Moreover, LAB can produce bacteriocins, attach to pathogen receptors on susceptible host cells through competitive exclusion, and inhibit the QS system [166,167,171]. The target microorganisms of LAB include Gram-positive bacteria (e.g., *Clostridium perfringens*, *Staphylococcus* spp., *Listeria* spp., *E. faecium*), Gram-negative bacteria (e.g., *E. coli*, *Salmonella typhimurium*, *Pseudomonas* spp., *Campylobacter* spp., *H. pylori*), as well as fungi (e.g., *Aspergillus niger*, *Aspergillus flavus*, *Penicillium roqueforti*) and viruses (e.g., rhinovirus, HIV—Human Immunodeficiency Virus, SARS-CoV-2—Severe Acute Respiratory Syndrome Coronavirus 2) [163,169,174,175,176]. In support of their antimicrobial properties, probiotics are considered valuable immune modulators by regulating the balance between pro- and anti-inflammatory cytokines [33,37,177,178]. In a recent study, we demonstrated that selected LAB strains (*Lactobacillus paracasei*, *Lactobacillus rhamnosus*), either alone or in combination with natural bioactive compounds including nerolidol, gallic acid, and α-terpineol, significantly modulated cytokine profiles by downregulating pro-inflammatory mediators (such as TNF-α and IL-6), while enhancing anti-inflammatory cytokines (including IL-10), highlighting their complementary potential in immune regulation [37].

Several LAB strains inhibited the in vitro adherence of bacterial pathogens to susceptible cells. Lactobacilli cells can adhere to each other and form aggregates, which generate barriers and prevent the adhesion of enteric pathogens [179]. *Lactobacillus plantarum* reduced the adhesion rate of *E. coli* to intestinal cells to 56% [180]. *Lactobacillus acidophilus* strains prevented the adhesion of *E. coli* to Caco-2 and HT-29 colon cancer cells [179]. Additionally, the adhesiveness of *E. coli* and *S. typhimurium* to porcine epithelial cells was reduced in the presence of *Lb. rhamnosus*, underlining the protective action exerted by LAB on intestinal barriers [177].

#### 4.2.2. Postbiotics and Probiotic Metabolites—Characterization and Mechanism of Action

**Figure 8:**
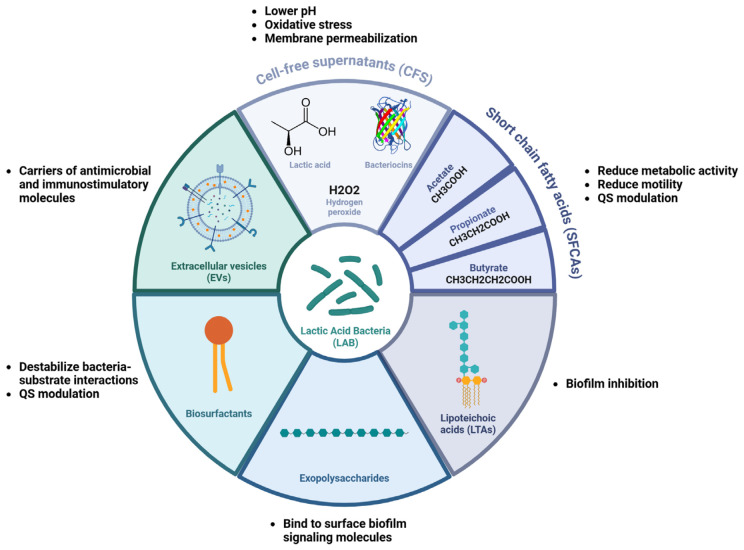
Representative postbiotics produced by LAB and their main mechanisms of action. Figure generated using biorender.com.

The term “postbiotics” has been defined as preparations of “inanimate microorganisms and/or their components that confer a health benefit on the host” [181]. Several postbiotics have been studied for their anti-biofilm action, including cell-free supernatants (CFS), short-chain fatty acids (SCFAs), cell wall fragments, exopolysaccharides, biosurfactants, and extracellular vesicles (EVs) [181,182,183,184,185] (Figure 8).

CFS represents the liquid phase of a probiotic culture, obtained after filtration to remove cells, which contains several metabolites [181,186,187,188,189]. It has been observed that cell-free formulations obtained from *Lb. plantarum* and *Bifidobacterium longum* cultures inhibited biofilm formation of MDR *E. coli* by 64.57% and 57.94%, respectively [190]. CFS of LAB are acidic due to the production of several organic acids, depending on the species and metabolic pathway; thus, the anti-biofilm efficiency of both acidic and pH-adjusted supernatants has been tested. *Lactobacillus casei* and *Lactobacillus reuteri* acidic CFS diluted by 1/16 reduced preformed biofilms of *Proteus mirabilis* by 60% and 73%, while they also blocked the initial bacterial adhesion by 72% and 73%. Neutralized CFS from *Lb. casei* and *Lb. reuteri* diluted by 1/16 inhibited biofilm formation by 39% and 73%, and adhesion by 75% and 73% [35]. CFS *from Lactobacillus fermentum*, *Lb. acidophilus*, *Lb. plantarum*, and *Lb. rhamnosus* inhibited adhesion and invasion of methicillin-susceptible *S. aureus* (MSSA) on human osteoblast cells, while also suppressing the formation of biofilms [36]. *Lb. plantarum* CFS were also proven to eradicate mature biofilms developed by *H. pylori* [191]. In the paper by Drumond et al., *Lactobacillus johnsonii* CFS was the most effective in inhibiting 85% of *P. aeruginosa* biofilms when added prior to biofilm formation and co-incubated compared with the preformed biofilm treatment (76% inhibition) [192]. Another study proved the biofilm eradication activities of CFS obtained from *Lb. plantarum* cultures against *S. aureus* (66.9%), *E. coli* (39.73%), coagulase-negative *Staphylococcus* (27.75%), and *P. aeruginosa* (7.85%) [166]. *Lactobacillus brevis*, *Lb. rhamnosus*, and *Lb. plantarum* CFS generated a notable anti-biofilm effect against Gram-positive pathogens, including *S. aureus* (MBEC = 50%) and *E. faecium* (MBEC = 0.2–0.39%) [33]. Overall, the anti-biofilm activity of LAB CFS is associated with the combined action of several postbiotic compounds, including lactic acid, H_2_O_2_, bacteriocins, and additional bioactive metabolites, as follows [181,186,187,188,189].

**Figure 9:**
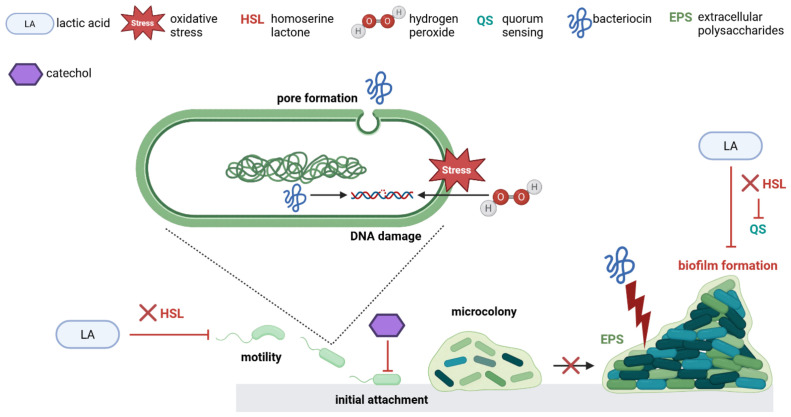
Mechanism of anti-biofilm action of LAB metabolites, as described above. Figure generated using biorender.com. Adapted with modifications from [188,196].

(1) Lactic acid is a hydroxycarboxylic acid, consisting of three carbon atoms, six hydrogen atoms, and three oxygen atoms [188]. Current evidence suggests that the bactericidal action of lactic acid is due to the generation of both intracellular and extracellular acidic conditions, damage to membrane integrity, and alteration of genomic DNA and QS signaling [193,194]. For example, lactic acid produced by a probiotic *Pediococcus acidilactici* strain inhibited swarming–swimming–twitching motility and QS by blocking the production of homoserine lactone (HSL), which led to the inhibition of biofilm formation in *P. aeruginosa* [194]. In addition, the growth and adhesion suppression generated by *Lb. rhamnosus* CFS on *P. aeruginosa* was mainly attributed to a CFS fraction with a molecular weight lower than 3 kDa, which mostly contained lactic acid [195] (Figure 9).
(2) H_2_O_2_ is an antimicrobial synthesized by several LAB strains, which causes oxidative stress, leading to DNA and cell damage and subsequent death [188,196] (Figure 9).
(3) Bacteriocins are AMPs synthesized in the ribosomes of Gram-positive bacteria, including LAB, as well as Gram-negative bacteria [186,187,188,189,197]. They possess a low molecular weight and broad-spectrum antibacterial and anti-biofilm mechanisms against various pathogens [171,188,189,198]. Lantibiotics are an example of well-characterized bacteriocins [197]. Their action against bacterial adhesion is justified by the interference of bacteriocins with the production of extracellular polysaccharides and the structural dissociation of bacterial membranes through the formation of pores, occurring both prior to and post-biofilm formation [186,188,189,196,198]. Pathogenic bacterial membranes are also permeable to bacteriocins, which leads to intracellular accumulation of bacteriocins, causing DNA damage. According to a recent comprehensive review by Pang et al., *Lactobacillus*-produced bacteriocins displayed anti-biofilm activities against *S. aureus*, *E. coli*, *Salmonella* spp., and *L. monocytogenes* [186]. Moreover, the bacteriocin nisin produced by *Lactococcus lactis* was described to inhibit biofilm development of *B. subtilis*, due to reduced EPS synthesis and bacterial surface hydrophobicity [183,199,200]. Thermophilin 110, a bacteriocin produced by a *Streptococcus thermophilus* strain, has displayed anti-*Listeria* spp. properties [200] (Figure 9).
(4) Catechol and caffeic acid are phenolic compounds with both antimicrobial and antioxidant activities; although they are not representative CFS constituents, but rather reported bioactive compounds, it has been noted that catechol attaches to the cell surface, preventing the initial bacterial attachment and development of biofilms [188] (Figure 9).

SCFAs comprise acetate, propionate, and butyrate produced during intestinal bacterial fermentation, which have been proven to impact bacterial virulence and biofilm development [188]. Butyrate and propionate modulated the QS mechanism, biofilm formation, and motility of *S. enterica* serovar *Typhimurium* (*S. Typhimurium*). Butyrate reduced the swimming motility in *S. Typhimurium*. Both SCFAs reduced the biofilm metabolic activity, while they also prevented pathogen adhesion and invasion into intestinal cells [201].

LTAs of Gram-positive bacteria are an example of cell wall fragments that possess biofilm inhibitory activities [181,187]. LTAs are amphiphilic glycolipids and major structural components of the *Lactobacillus* cell wall, contributing to bacterial proliferation, surface adhesion, and biofilm formation [187]. LTAs from four *Lb. plantarum* strains had strain-dependent effects on dental pathogens such as *E. faecalis*, *Streptococcus gordonii,* and *S. mutans*. *Lb. plantarum* LRCC 5310 LTA was the most effective against biofilms generated by all pathogens, suggesting the potential use of this cell wall component for the prevention of dental infections [202].

Exopolysaccharides are high-molecular-weight, long-chain polymers synthesized during LAB growth [181,183,189,198]. The anti-biofilm effect of exopolysaccharides can be justified by their in vitro interference with cellular communication by binding to surface biofilm signaling molecules or glycocalyx receptors of bacterial pathogens [183,189,198]. The anti-biofilm properties of exopolysaccharides produced by several *Lactobacillus* species have been demonstrated against an array of Gram-positive and Gram-negative pathogens [203]. In addition, the paper by Tarannum et al. shed light on the multiple bioactive properties of *Lb. fermentum* exopolysaccharides, such as antioxidant, anti-inflammatory, antimicrobial, and anti-biofilm. The exopolysaccharides were more effective in mitigating biofilms produced by *S. aureus* (76.61%) compared with *E. coli* (13.64%) [204].

Biosurfactants are amphiphilic compounds that prevent bacterial adhesion through LPS binding and reduce the hydrophobic character of the cell surface [183,196]. Biosurfactants, given their amphiphilic nature, can prevent initial adhesion through altering the forces stabilizing the interactions between the bacteria and the substrate; in addition, these compounds have been reported to interfere with the QS network and dissociate established biofilms in several bacterial models [182,183,205,206]. In this sense, the QS-inhibitory action of the *Lb. rhamnosus* biosurfactant has been demonstrated by perturbation of HSL and violacein in *Chromobacterium violaceum*, and in *P. aeruginosa*, by reducing pyocyanin levels. Exopolysaccharide synthesis and swarming motility were suppressed in *C. violaceum* (61.13%) and *P. aeruginosa* (53.11%), with an overall biofilm formation inhibition of 68.12% and 59.80%, respectively [205]. In a similar manner, *Lb. acidophilus* biosurfactants were anti-virulent since they mitigated the synthesis of violacein in *C. violaceum*, prodigiosin in *Serratia marcescens,* and pyocyanin in *P. aeruginosa*. The biosurfactants also reduced exopolysaccharides production, swimming motility, as well as biofilm formation [207]. Additionally, *Lb. rhamnosus* biosurfactants were reported to disrupt preformed biofilms of *S. aureus* (55.77%), *P. aeruginosa* (59.78%), *E. coli* (66.65%), and *B. subtilis* (70.49%) at concentrations between 12.5–50 mg/mL [206].

Extracellular vesicles (EVs), increasingly recognized as components of postbiotics, are considered contributors to the inhibitory activity associated with probiotic strains [184,208,209]. EVs are produced by prokaryotic cells, including probiotics, as well as eukaryotic cells and comprise spherical lipidic particles with a size of 20–400 nm [185,208,209,210,211]. Initially, Gram-negative bacteria were considered the sole producers of EVs, considering the structural resemblance of EVs to the outer membrane, while EVs have also been identified in Gram-positive microorganisms, including LAB (*Lb. plantarum*, *Lb. paracasei,* and *Lb. rhamnosus*) [33,184,212,213,214,215]. EVs released by LAB support host health by being carriers of bioactive molecules with antimicrobial and immunostimulatory properties [184,209,211,212,213,216,217]. By the delivery of these compounds, coupled with competitive exclusion of pathogens, EVs from *Lactobacillus* strains can hinder adhesion of opportunistic bacteria to susceptible host cells [184]. EVs derived from probiotic bacteria have demonstrated activity against a range of microorganisms, including both Gram-positive and Gram-negative species, as well as biofilm-forming pathogens. In this sense, the antibacterial properties of EVs have been previously studied, displaying inhibitory activities against pathogens including *Shewanella putrefaciens*, *Propionibacterium acnes*, and *S. aureus*. In addition to direct antimicrobial effects, EVs can modulate host immune responses, including the regulation of cytokine production, such as promoting the release of anti-inflammatory cytokines and blocking the synthesis of pro-inflammatory cytokines [184,213,214,218,219,220]. Collectively, this evidence highlights EVs as multifaceted postbiotics with potential relevance in both antimicrobial and anti-biofilm strategies.

### 4.3. Classes of Natural Anti-Biofilm Compounds (Terpenoids, Phenolic Acids, Flavonoids, Alkaloids)

In recent years, plant-derived bioactive compounds have gained attention due to their promising inhibitory effects on bacterial pathogenesis and biofilm development. The therapeutic potential of medicinal plants is largely attributed to their rich repertoire of secondary metabolites, which exhibit significant chemical and structural diversity and are responsible for a wide range of biological activities. The use of medicinal plants has historically been rooted in traditional therapeutic practices, and contemporary research has increasingly validated this empirical knowledge by elucidating the molecular mechanisms underlying their bioactivity, thereby confirming their role as a major source of pharmacologically active agents [221,222,223].

Indeed, more than 80% of currently available drugs are either directly derived from or inspired by natural compounds. However, despite their extensive biological potential, only a limited number of phytochemicals such as capsaicin, codeine, colchicine, paclitaxel, and reserpine have received regulatory approval for clinical use by the U.S. Food and Drug Administration (FDA) [38,222,224].

Phytochemicals, also known as phytobiotics, phytogenics, or botanicals, can be broadly classified into two categories: primary plant metabolites, such as carbohydrates, lipids, proteins, vitamins, enzymes, and pigments, which are essential for basic cellular functions, and secondary plant metabolites, which play crucial roles in plant adaptability and survival [39,225].

These secondary metabolites contribute to defense against pathogens and environmental stressors. Among these secondary metabolites, six major groups of phytochemicals have been recognized for their significant anti-biofilm potential, highlighting their relevance as alternative or adjuvant therapeutic agents for combating BAIs: alkaloids, terpenes, flavonoids, saponins, phenolic acids, tannins, and volatile oils (Table 3) [38,226].

Furthermore, plant-derived bioactive compounds exhibit remarkable structural diversity, which supports their broad spectrum of pharmacological properties. In addition to their anti-biofilm activity, these compounds display antioxidant, immunomodulatory, anxiolytic, larvicidal, and anticancer effects through multiple, often complementary, mechanisms [4,37,222,227]. Moreover, the incorporation of natural bioactive compounds into dietary supplements, functional foods, and cosmeceutical products highlights their growing relevance in preventive healthcare strategies and wellness products [223].

**Table 3:** Overview of natural bioactive compounds classes, their chemical characteristics, biofilm-associated target pathogens, and mechanisms of anti-biofilm action.

Class of Natural Compound	Representative Examples	Major Targeted Biofilm-Forming Pathogens	Anti-Biofilm Strategies	References
Alkaloids *Organic heterocyclic nitrogen derivatives* (*amines*)	Caffeine, capsaicin, quinine, berberine, piperine, hordenine, solanine, tomatine	*S. aureus* (including MRSA) *S. mutans* *E. faecalis* *Listeria* spp. *Bacillus* spp. *E. coli* *P. aeruginosa*	Efflux pumps inhibition; Suppression of DNA replication, transcription, and translation; Disruption of QS systems by downregulating QS-related genes (involved in AIs synthesis, biofilm maturation, and motility); Inhibition of virulence factor expressions (including intercellular adhesion-associated genes) and key enzymatic activity; Reduction of biofilm-associated metabolites leading to disintegration of biofilm integrity; Impairment of adhesion and EPS production; Destabilization of cell membrane integrity.	[27,28,190,192,193,194,195,196,197,198]
Terpenoids/Terpenes (isoprenoids) *Natural isoprenoid derivatives*	Monoterpenes: 53% camphor, eucalyptol, limonene, linalool, terpineol, α- and β-pinene Diterpenes: 1% retinal, retinol, phytol, carnosic acid; Triterpenes: squalene, lanosterol, oleanol, betulin Tetraterpenes: lycopene, a- and b-carotenoids, zeaxanthin Polyterpenes: 18% natural rubber—cis-polyisoprene, gutta-percha, natural latex	*S. aureus* (including MRSA) *S. typhimurium* *E. coli* *B. cereus* *P. aeruginosa* *Streptococcus pyogenes*	Membrane destabilization mediated by the lipophilic components; Inhibition of virulence factor expression; Increased membrane permeabilization; EPS matrix degradation; Interference with QS pathways; Suppression of genes involved in biofilm regulation; Eradication of pre-established biofilms.	[27,28,190,198,199]
Flavonoids *Polyphenolic compounds*	Flavones: luteolin, diosmetin, chrysin, apigenin Flavonols: quercetin, myricetin, rutin, kaempferol Flavanones: hesperetin, naringenin Flavanols: catechin, epicatechin, epigallocatechin Isoflavones: daidzin, genistein Anthocyanins cyanidin, peonidin	*S. aureus* *E. coli* *P. aeruginosa* *H. pylori*	Modulation of QS pathways → suppression of biofilm development, reduced motility, decreased EPS production, and downregulation of virulence-associated phenotypes; Repression of the psIA gene → reduced the synthesis of AHL, interfering with the QS system; EPS matrix degradation; Prevention of microbial adhesion to biotic and abiotic surfaces; Inhibition of efflux pump systems.	[27,28,190,198,199,200]
Phenolic acids *Aromatic derivatives with a hydroxyl or carboxyl group*	Cinnamic acid, benzoic acid, ferulic acid, coumaric acid, caffeic acid, salicylic acid, gallic acid, p-hydroxybenzoic acid	*S. aureus* *S. mutans* *E. coli* *P. aeruginosa* *L. monocytogenes* *P. mirabilis*	Inhibition of QS through reduced AIs production, disrupting microbial intercellular communication; Repression of EPS synthesis and biofilm matrix assembly; Membrane destabilization via alterations in membrane potential; Metal ion chelation (e.g., Ca^2+^, Fe^2+^, /Fe^3+^) impairing enzymatic and metabolic functions; Attenuation of microbial adhesion through upregulation of the regulatory gene icaR; Suppression of virulence-associated gene expression (gtfB, gtfC, gtfD) involved in biofilm development and pathogenicity.	[27,28,190,198,199,200,201,202,203,204]
Saponins *Chemical derivatives that contain a sapogenin compound*	Triterpenoid saponins: ginsenosides, glycyrrhizin, asiaticoside, aescin Steroidal saponins: dioscin, protodioscin, glycyrrhizin	*S. aureus* *E. coli* *Cutibacterium acnes* *Candida albicans* *P. aeruginosa* PAO1	Prevention of cell aggregation, surface attachment, and biofilm formation; Repression of EPS biosynthesis; Downregulation of genes involved in biofilm development and maintenance; Anti-QS activity; Inhibition of virulence factor production; Disruption of cell membrane integrity.	[28,190,205,206,207,208,209,210,211,212]
Tannins *High molecular weight polyphenols*	Condensed tannins: tannic acid, ellagic acid Hydrolysable tannins: epigallocatechin gallate—EGCG Complex tannins: castalagin, vescalagin	*Staphylococcus* spp. *Pseudomonas* spp. *Streptococcus* spp. *S. typhimurium* *E. coli*	Inactivation of microbial adhesins, disruption of enzymes, and biosynthesis of cell wall-associated proteins; Downregulation of attachment-related genes (agrA, icaA, icaD); Interference with QS regulatory RNAs; Inhibition of efflux pump systems; Disruption of cell membrane integrity.	[27,28,190,199,213,214]

### 4.4. Mechanisms Underlying the Anti-Biofilm Effects of Natural Compounds

Plant-derived secondary metabolites, including phenolic compounds (such as phenolic acids, quinones, flavonoids, flavones, flavonols, tannins, and coumarins) as well as essential oils, terpenoids, lectins, alkaloids, polypeptides, and polyacetylenes, exhibit strong anti-biofilm activity through multiple complementary mechanisms. These compounds interfere with key stages of biofilm development by inducing substrate deprivation, disrupting cell membranes, and interacting with essential cellular targets, including proteins and nucleic acids [24].

**Figure 10:**
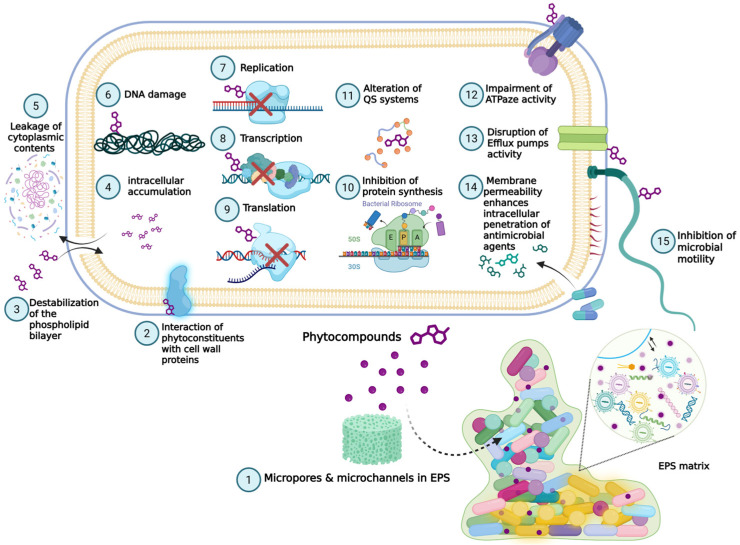
Major anti-biofilm mechanisms of phytoconstituents. Plant-derived compounds exert their effects through distinct modes of action, collectively influencing biofilm architecture, microbial cell structure, and key metabolic and regulatory processes, as illustrated in panels (**1**–**15**) and described above. Figure generated using biorender.com. Adapted with modifications from [227].

At the molecular level, phytocompounds penetrate the biofilm matrix and induce structural alterations by forming microchannels and micropores within the EPS matrix, thereby facilitating their diffusion throughout the biofilm (Figure 10(1)). Once internalized, these compounds exert their effect by damaging the cell wall and interacting with bacterial cell wall proteins (Figure 10(2)), thereby destabilizing the phospholipid bilayer and increasing membrane permeability (Figure 10(3)) [228]. This process leads to intracellular accumulation of bioactive molecules [229] (Figure 10(4)), leakage of cytoplasmic contents (Figure 10(5)), particularly ions, disruption of essential membrane functions, and ultimately cell lysis and death by affecting multiple biological processes such as gene regulation, ionic balance, and altering signaling pathways [230].

In parallel, phytochemicals can lead to alteration of DNA and RNA and inhibit key enzymatic processes involved in DNA replication, transcription, and translation (Figure 10(6–10)). These effects result in the suppression of gene expression, intracellular metabolism, and cellular proliferation. Their interference with translation and the consequent inhibition of protein synthesis further reduces EPS production [231], virulence factor expression, and QS autoinducer synthesis, compromising QS systems (Figure 10(11)) and ATPase activity [232,233,234] (Figure 10(12)), thereby impairing cell coordination and consequently biofilm maturation and community organization.

Moreover, plant-derived compounds display significant anti-biofilm effects by interfering with microbial adhesion through surface anti-adhesive mechanisms, thereby preventing early biofilm formation and decreasing the risk of BAIs [22,227]. Furthermore, natural compounds can inhibit efflux pump systems by disrupting proton gradients, leading to impaired efflux pump activity and enhanced intracellular retention of antimicrobial substances [232,235] (Figure 10(13)). In addition, increased membrane permeability facilitates intracellular penetration of antimicrobial agents (Figure 10(14)). Additionally, by repressing genes encoding structural components of pili, flagella, and curli (Figure 10(15)), phytochemicals reduce microbial motility, intercellular aggregation, and biofilm dispersion [24,227,236].

Collectively, these multifaceted mechanisms underscore the strong potential of plant-derived compounds as effective anti-biofilm agents, targeting both the biofilm matrix and embedded microbial cells, and thus contributing to the prevention of biofilm formation and the eradication of mature biofilms. In addition, their generally low cytotoxicity, high consumer acceptability, and ability to overcome or limit AMR make them attractive candidates for applications in the food industry, topical formulations, and oral hygiene products [4].

The anti-biofilm activity of natural compounds can be conceptualized as a hierarchical, multilevel process that involves coordinated actions at the matrix, cellular, and regulatory levels of biofilm organization (Table 4).

a. Matrix-level anti-biofilm mechanisms

At the matrix level, plant-derived compounds primarily target the structural integrity of the biofilm by disrupting the EPS. These compounds can penetrate the biofilm matrix and induce structural alterations by forming microchannels and micropores within the EPS, thereby facilitating their diffusion throughout the biofilm. Additionally, several phytochemicals reduce EPS and alginate production, leading to impaired matrix assembly and decreased biofilm stability [22,227,228]. These effects collectively weaken the physical barrier function of the biofilm and enhance the susceptibility of embedded cells.

Moreover, phytochemicals interfere with microbial adhesion, which represents the initial and critical step of biofilm formation, by inhibiting the synthesis and expression of adhesins and surface-associated proteins. This effect is mediated, in part, through the downregulation of genes involved in adhesion processes, such as the *ica* operon, thereby preventing surface attachment in early biofilm development [237].

b. Cellular anti-biofilm mechanisms

At the cellular level, phytochemicals directly compromise microbial cell integrity and metabolism. They interact with microbial cell walls and membranes, destabilizing the phospholipid bilayer and increasing membrane permeability. This leads to intracellular accumulation of bioactive compounds, leakage of cytoplasmic contents, disruption of proton gradients, and impairment of efflux pump systems. Additionally, several compounds inhibit key metabolic enzymes and ATPase activity, thereby reducing energy production and promoting cell death [228,229,232,235]. Moreover, several phytochemicals directly interact with nucleic acids and key intracellular enzymes, inducing DNA and RNA damage, and inhibiting essential processes such as DNA replication, transcription, and translation. These effects suppress intracellular metabolism, inhibit cellular proliferation and division [231].

c. Regulatory anti-biofilm mechanisms

At the regulatory level, natural compounds modulate genetic and signaling pathways that coordinate biofilm development and community behavior. Inhibition of QS systems by suppressing AIs production and blocking AIs-receptor interactions downregulates QS-controlled traits such as virulence factor expression, EPS biosynthesis, and motility, thereby impairing biofilm maturation and stability [232,233,234].

Despite the large number of phytochemicals reported to inhibit biofilm formation, only a limited subset has been investigated at the molecular level, with most studies restricted to phenotypic observations such as reduced biomass or EPS production (Table 4).

The antimicrobial and anti-biofilm activity of natural bioactive compounds against biofilm-forming pathogens is commonly quantified using four parameters: MIC, MBC, MBIC, and MBEC. MIC and MBC values constitute valuable parameters that provide insight into the intrinsic antimicrobial potency of the investigated phytochemicals against planktonic cells, which serves as a relevant reference framework for interpreting their anti-biofilm activity. Given that biofilm-associated microorganisms display markedly enhanced tolerance compared with their planktonic counterparts, comparison of MIC/MBC with MBIC/MBEC values enables assessment of the concentration required to inhibit biofilm formation or eradicate established biofilms. The inclusion of both planktonic and biofilm-related parameters facilitates a more comprehensive characterization of the antimicrobial profile of each compound [238].

Representative anti-biofilm phytoconstituents, their corresponding antimicrobial and anti-biofilm activity values, and their principal mechanisms of action are summarized in Table 4.

**Table 4:** Anti-biofilm activity, effective concentrations, and mechanisms of action of natural bioactive compounds against clinically relevant pathogens.

	Compound	Pathogen Targeted	MIC/MBC/MBIC/MBEC	Anti-Biofilm Mechanism of Action	References
Matrix-level anti-biofilm mechanisms	Naringin	*P. aeruginosa*	MIC = 128 μg/mL MBC = 128 μg/mL	Inhibition of EPS and alginate production; Disruption of mature biofilm.	[239]
	Quercetin	*S. epidermidis* ATCC 35984	MBIC = 0.25–0.5 mg/mL	Decreased the synthesis of EPS and altered the composition.	[240]
	Thymol	*S. aureus* MRSA TCH1516	MBIC = 64 μg/mL	Reduced the production of intracellular adhesins and eDNA released.	[241]
	Tea catechin epigallocatechin gallate	*P. gingivalis* 381	MBIC = 500 mg L^−1^	Inhibition of the initial adhesion stage and eradication of established biofilms; disruption of the cell wall and cell membrane by production of hydrogen peroxide within the phospholipid bilayer.	[242]
	Quercetin	*S. aureus* strains (C104, V858, newman-bap)	MBIC = 10 μg/mL	Prevention of the assembly of Biofilm-associated proteins (Bap)-related amyloid-like structures leading to inhibition of biofilm development.	[243]
	Myricetin		MBIC = 10 μg/mL		
	Scutellarein		MBIC = 5 μg/mL		
	Epigallocatechin gallate	*Shigella flexneri* ATCC 12022	MBIC = 50 μg/mL	Suppression of the *mdoH* gene, preventing the EPS production.	[244]
Cellular-level anti-biofilm mechanisms	Rosmarinic acid	*C. albicans*	MBEC = 0.4 mg/mL	EPS matrix degradation; decreased EPS production; Reduced mitochondrial activity; Inhibiting the production of enzymes.	[245]
	Geraniol (>98% HPLC purity)	*S. aureus* MRSA ATCC	MBIC = 256 μg/mL	Decreased eDNA release, inhibited the synthesis of intracellular adhesins, staphyloxanthin production; downregulation of genes involved in virulence factor production and biofilm development (*sarA*, *fnbA*, *fnbB*, *clfA*, *icaA*, *icaB*, *atlA*, *crtM*).	[246]
	Ellagic acid	*E. coli* BW25113	MBIC = 50 μM	Inhibition of the enzyme function of WrbA, which is involved in biofilm formation, modulation, and ROS release.	[247]
	Gallic acid	*S. aureus*	MBIC = 2 mg/mL	Regulating the expression of the *ica* operon—suppression of *icaA* and *icaD* genes (leading to decreased adhesin expression and impaired EPS synthesis).	[248]
Regulation-level anti-biofilm mechanisms	Hordenine	*P. aeruginosa*	MIC = 2.5 mg/mL	Suppression of QS regulatory genes *lasI*, *lasR*, *rhlI*, *rhlR*; Inhibition of HSL production, leading to disruption of intercellular communication.	[249]
	Hesperidin	*S. aureus*	MBIC = 100 μg/mL	Downregulation of biofilm-associated gene *sarA*, fibronectin-binding protein *fnbA*, *fnbB*, staphyloxanthin production *crtM*, autolysin *altA*, polysaccharide intracellular adhesion gene *icaD*, *icaA*.	[250]
	Curcumin (95% from turmeric rhizome)	*P. aeruginosa* PA14 *B. subtilis* ATCC 6051	Conc. of 2.5 to 5 μg/mL	Anti-QS activity by downregulating *lasI/lasR* systems and *luxS*/AI-2 system.	[251]
	Berberine	*S. aureus* MRSA	MBIC = 32 μg/mL	Downregulation of the key dispersal regulator *agrA* leads to impaired biofilm dispersal.	[252]
N.D. not determined	Nerolidol	*E. faecalis* 188 (isolated from Hidradenitis Suppurativa lesions)	MBEC = 0.25 mg/mL	Nerolidol may disrupt or eradicate established biofilms due to its hydrophobic nature, the length of its aliphatic chain, and the presence of a hydroxyl functional group. Furthermore, a decrease in EPS production has been observed.	[37]
	Nerolidol	*P. aeruginosa* LMB303 *S. aureus* LMB403 *S. aureus* LMB409 *K. pneumoniae* LMB309	Conc. of 0.5 to 4 mg/mL		[221,253]
	Gallic acid	*P. aeruginosa* CIP4.83 *S. aureus* CIP103.467	MBEC = 0.5 mg/mL (efficient on *P. aeruginosa* biofilm)	Gallic acid potentially disrupts the QS system by suppressing AHL synthesis and increasing membrane permeability, which promotes Ca efflux, intracellular content leakage, and cell death; its ability to chelate metal ions (e.g., Ca and Fe) may disrupt membrane protein function and cellular metabolism. Carvacrol may permeabilize and depolarize microbial membranes, leading to the leakage of intracellular contents and eventual cell death.	[230]
	Curcumin		Conc. of 0.5 mg/mL—no eradication properties		
	Carvacrol		MBEC = 0.5 mg/mL (efficient on *S. aureus* biofilm)		
	Carvacrol	*E. coli*	MBIC = 0.6 mg/mL; MBIC = 1.2 mg/mL	Potential mechanisms include disruption of QS systems and interference with EPS production.	[254]

### 4.5. Synergism Between Natural Bioactive Compounds and/or Antibiotics/Antimicrobial Strategies

#### 4.5.1. Synergistic Interactions Among Natural Bioactive Compounds

The global health burden exerted by the dissemination of MDR bacterial strains and the consequent persistent infections as a result of biofilm formation, especially on medical devices, reinforces the need to implement novel anti-biofilm strategies [33,43].

In recent years, increasing attention has been directed toward synergistic combinations of natural and synthetic therapeutic agents as a strategy to improve anti-biofilm efficacy [255]. Synergy refers to the interaction between two or more agents that results in a combined effect greater than the sum of their individual effects. Synergistic antimicrobial effects might be due to mechanisms such as increased membrane permeability, targeting of multiple cellular pathways, or enhancement of antimicrobial uptake.

Several studies have explored synergistic interactions involving lactoferrin and whole LAB cells, as well as their metabolites, such as bacteriocins or CFS. For instance, the prebiotic activity of Lf on LAB strains, as well as the antimicrobial action of the bLf-LAB combination, has been reported, underlining a notable growth inhibition effect [126,173]. In combination with nisin, Lf improved the activity of this bacteriocin against Gram-negative bacteria. Lf could increase the permeability of the bacterial outer membrane and cell wall, so nisin could penetrate the inner membrane. The combination of 500 μg/mL Lf and 250 IU/mL of nisin was effective against *E. coli*, while 500 μg/mL Lf and 10 IU/mL of nisin inhibited the multiplication of *L. monocytogenes* [256]. Additionally, the bLf/nisin mixture inhibited *P. aeruginosa* biofilms by 79.70%, compared with the inhibition caused by the compounds individually (58.02% and 30.34%, respectively). The combined treatment also reduced pyocyanin production to 0.85 µg/mL, as opposed to the production of pyocyanin in the presence of bLf (1.84 µg/mL) and nisin (2.62 µg/mL) alone. The results might indicate an anti-virulence property of the bLf/nisin treatment, explained by the destabilization of virulence pathways mediated by QS mechanisms [40].

In line with these observations, our recently published preliminary study aimed to evaluate the inhibitory action of purified bLf, commercial food supplement-derived Lf, alone or combined with CFS obtained from *Lb. rhamnosus*, *Lb. brevis*, and *Lb. plantarum* cultures, against the growth and adhesion of several opportunistic bacterial strains. With respect to the effect of the combination of commercial Lf and LAB CFS on adhesion to inert substrates, the anti-biofilm activity was greater, especially against Gram-positive pathogens. In fact, a complementary effect between Lf and CFS was observed, as the concentration of Lf required to achieve an inhibitory effect was significantly lower when CFS was added compared with Lf individually. These results support the potential applications of Lf and CFS-based decontamination methods of biofilms developed on medical devices, thus limiting persistent infections. In terms of bacterial adhesion to the cellular substrate comprising HCT-8 human intestinal cells, the anti-adhesion effect of Lf was potentiated by the addition of LAB CFS, while the combination of the CFS of *Lb. rhamnosus* and Lf exerted an enhanced effect. These results suggest the potential prophylactic and therapeutic use of Lf and LAB CFS, considering their ability to hinder pathogen adhesion to susceptible host cells, preventing the onset of an infection [33]. Similarly, a previous paper by Chen et al. also highlighted the synergism between apo-bLf and *Lb. fermentum* supernatants against the growth of methicillin-resistant *S. aureus* (MRSA) [126]. The underlying mechanisms are likely multifactorial and include membrane interactions, iron sequestration by Lf, and the activity of CFS-derived bioactive compounds, such as organic acids, bacteriocins, and other antimicrobial metabolites. These compounds can exert their effects on the cell surface or inside the biofilm matrix, without necessarily requiring intracellular penetration. These findings support the potential of Lf-based combinations as complementary strategies to enhance antimicrobial and anti-biofilm activity.

The use of LAB-derived fractions such as CFS, cellular suspensions (CS), or cellular lysates, in combination with natural bioactive compounds, is an emerging and highly promising strategy in wound healing applications. These LAB-based systems are rich in bioactive molecules, which exhibit antimicrobial, anti-biofilm, and immunomodulatory properties. The use of LAB-derived supernatants and cellular lysates represents a safer and more stable alternative to viable probiotics, particularly in wound healing applications where the use of live bacteria may be limited. In parallel, plant-derived bioactive compounds are widely recognized for their anti-inflammatory and antioxidant properties. The combination of LAB-fractions and plant-derived compounds may act synergistically, particularly in the context of chronic wounds, where biofilm formation and persistent inflammation impair the healing process, with each component potentially acting as an adjuvant to enhance the overall therapeutic effect. Available evidence suggests that this bidirectional interaction could represent an effective approach for modulating skin microbiota, improving infection control, promoting tissue regeneration processes, including fibroblast proliferation, collagen deposition, and angiogenesis. For instance, Kaur et al. reported that a wound dressing incorporating *Lb. plantarum* UBLP-40 (MTCC 5380) and curcumin-loaded nanoparticles significantly reduced biofilm formation of *S. aureus* 9144 and accelerated wound closure, while decreasing levels of TNF-α, matrix metalloproteinase-9 (MMP-9), and lipid peroxidation (LPO) [41].

Similarly, another recent study demonstrated that the combination of *Lb. rhamnosus*-derived CFS and sandalwood essential oil at sub-inhibitory concentrations exhibited anti-adhesion effects against both Gram-positive (*S. aureus*) and Gram-negative (*Morganella morganii*) strains. This suggests a potential adjuvant role of LAB-derived fractions and natural resources in reducing bacterial adhesion and preventing early-stage biofilm formation [257].

By combining multiple LAB strains, it is presumed that they can potentiate each other’s beneficial actions or antimicrobial mechanisms. Thus, the inhibitory action of various LAB combinations has been evaluated against MDR *Staphylococcus* isolates. The *Lb. acidophilus*, *Lb. casei* and *Lb. plantarum* consortium inhibited the growth of these isolates by 85%, exhibiting a higher antimicrobial effect compared with LAB strains individually [258].

Organic acids used as antimicrobials with common applications in food preservation include lactic acid, acetic acid, citric acid, butyric acid, and malic acid. The individual organic acids, as well as their combinations, reduced *E. coli* biofilm cell viability by 90%. The most efficient antimicrobial action was noted in the case of the mixture between three organic acids: lactic, acetic, and either malic, citric, or butyric acid, compared with the lactic-acetic acid treatment. The three-acid combination also induced a better cell membrane permeabilization and intracellular contents leakage as opposed to the acids alone [259].

However, despite their promising potential, studies investigating such combinations remain limited, highlighting the need for further research to elucidate their mechanisms of action and optimize their therapeutic applications.

Given the structural and functional heterogeneity of biofilms, the combination of phytoconstituents that exert distinct anti-biofilm mechanisms is increasingly being investigated as a strategy to achieve effective biofilm control and eradication. The combined use of natural compounds has been shown to enhance anti-biofilm efficacy by targeting both early-stage adhesion processes and disrupting sessile cells within established biofilms. This supports the concept that the biological activity of plant extracts arises primarily from complementary, potentially synergistic interactions among their constituent molecules rather than from the action of individual compounds. Such interactions highlight their potential as novel anti-biofilm agents or adjuvants to conventional antimicrobial therapies [38,223,260,261].

The Fractional Inhibitory Concentration Index (FICI) is widely used as a quantitative parameter for evaluating interactions between antimicrobial agents. Based on FICI values, interactions are commonly classified as synergistic (S; FICI ≤ 0.5), additive (or independent interaction) (Ad; 0.5 < FICI ≤ 4.0), or antagonistic (FICI ≥ 4.0) [38,262]. However, it should be noted that the reference thresholds used for the interpretation of these interaction categories may vary across studies, depending on the experimental design and the criteria adopted by different authors, reflecting methodological variability.

To determine the FICI, the fractional inhibitory concentration (FIC) of each compound must first be calculated. For two compounds, the FIC is defined as the ratio between their MIC in combination and their MIC when used alone. The overall FICI is obtained by summing the FIC values of both compounds, providing a quantitative measure of their interaction.

Additionally, fractional bactericidal concentration (FBC) is calculated using the same approach as FIC, by replacing MIC values with MBC values. The resulting fractional bactericidal concentration index (FBCI) is interpreted using the same interaction criteria as FICI, classifying interactions as synergistic, additive, or antagonistic [42,262,263] (https://www.eucast.org/clinical_breakpoints/, accessed on 26 January 2026).

Table 5 summarizes combinations of natural bioactive compounds with anti-biofilm activity against clinically relevant pathogens, including the concentrations used to inhibit or eradicate biofilms, their combined effects, and potential mechanisms of action.

#### 4.5.2. Synergistic Combinations Between Natural Bioactive Compounds and Antibiotics/Antimicrobial Strategies

Considering the potential of Lf and Lf-derived AMPs to be used as prophylactic agents, the research shifted to the use of this iron-chelating glycoprotein in association with antibiotics to manage MDR infections [43,126]. The observed synergism or complementary action was explained primarily by the increased membrane permeability for antibiotics under the action of Lf or Lf AMPs, or by antibiotics promoting AMP binding to the outer membrane [139,264]. Early studies demonstrated the ability of LFcinB to enhance the bactericidal action of erythromycin in *E. coli* [139]. A synthetic hLf-derived peptide, hLf1–11, shifted the phenotype of *K. pneumoniae* strains to be susceptible to hydrophobic antibiotics, which are usually ineffective against Gram-negative pathogens since they are unable to penetrate the outer membrane. The data further revealed a synergistic effect between hLf1–11 and clindamycin, rifampicin, and clarithromycin against *K. pneumoniae*, reducing their MIC by 64-fold [265]. The report by Al-Mogbel et al. highlighted the additive effect of bLf alongside conventional antibiotics, which determined several phenotypic shifts, such as MRSA turning MSSA, quinolone-resistant strains turning susceptible, and vancomycin-resistant Enterococci (VRE) turning susceptible. These results reinforce the use of Lf as an adjuvant in novel strategies to reduce the incidence of MDR infections [116]. LFcinB was reported to even enhance the inhibitory effects of antibiotics such as amoxicillin and clindamycin against obligate and facultative anaerobic biofilms responsible for chronic oral infections [266].

Growing evidence indicates that co-administration of natural bioactive molecules with conventional antibiotics significantly enhances antimicrobial properties by increasing drug effectiveness and mitigating resistance mechanisms. In this context, phytoconstituents act as antibiotic potentiators [230]. These enhanced effects are frequently attributed to complementary, multi-target mechanisms, whereby antibiotics predominantly interfere with essential intracellular processes such as DNA replication or protein synthesis, while natural compounds disrupt biofilm integrity and QS systems. This multimodal strategy represents a promising approach for improving the management of BAIs [267]. In such synergistic interactions, one agent facilitates or reinforces the primary mode of action of another, ultimately resulting in superior therapeutic activity, particularly when antibiotics are combined with natural bioactive compounds capable of partially or completely suppressing resistance mechanisms [268].

Table 6 summarizes combinations between natural bioactive compounds and antibiotics with inhibitory activities against relevant pathogens, alongside their combined effects and potential mechanisms of action.

Novel formulations, in the form of hydrogels packaged with lactoferrin-dsiRNA-silver nanoparticles (AgNPs), have been considered for the clinical management of diabetic foot infections [278]. The evolution of a chronic wound infection relies on the development of biofilms by contaminating bacteria [279]. RhLf, in the formulation of hydrogels packaged with NPs, reduced *S. aureus* biofilms by ~70%, and those of *P. aeruginosa* by ~80% [278]. A hydrogel packaged with 2% Lf and 5% xylitol combined with a commercially available silver wound dressing induced a 4.7 log reduction in *P. aeruginosa* biofilm viability compared with the dressing alone (3.6 log reduction) [279]. Gelatin-based hydrogels coupled with AgNPs functionalized with Lf were proposed as wound dressings, given their strong anti-biofilm effects against *P. aeruginosa* and *S. aureus* [280].

Furthermore, to ameliorate the challenge represented by phage resistance, staphylococcal phages have been coupled with Lf or LAB strains. The association between *Lb. rhamnosus* and bacteriophage SA11 restricted *S. aureus* growth by 4 log under simulated intestinal secretions, supporting the combined use in preventing enteric bacterial infections [281]. By combining Lf with bacteriophages, biofilm biomass and metabolism were reduced, and the re-establishment of biofilms was also prevented [156].

The association between QQ enzymes and antimicrobials is of interest in current anti-infective therapies. Therefore, the potential synergism between Lf AMPs and QQ enzymes such as hexahistidine-containing organophosphorus hydrolase (His_6_-OPH) was explored, revealing an additive effect. The degradation of His_6_-OPH substrates, including HSL, was promoted in the presence of LFcinB. The efficacy of LFcinB against Gram-negative bacteria like *B. subtilis* was augmented in the presence of His_6_-OPH, since this enzyme hydrolyzes lactone-containing biofilm signaling mediators. Lastly, the potential co-administration of His_6_-OPH/LFcinB and probiotics was suggested, given the reduced antibacterial effect of the AMP-enzyme combination on *Lactobacillus* spp. The joint use of His_6_-OPH/LFcinB could also be extended, besides prophylaxis and treatment of MDR infections, to treat surfaces or in the food industry [282].

Lf and lysozyme count among the innate immune effectors ensuring the protection of mucosa by their lytic action against pathogens and are located in similar host niches; therefore, various studies aimed to evaluate their potential synergistic action [283,284]. Evidence suggests a synergistic effect between Lf and lysozyme, which may be attributed to the ability of Lf to interact with bacterial cell wall components, thereby facilitating the bioactivity of lysozyme. Within this framework, the synergistic action of these proteins against Gram-negative bacteria can be attributed to Lf permeabilizing the outer membrane and allowing lysozyme to cleave the peptidoglycan [283,285]. For example, Lf and lysozyme synergistically induced a lethal effect on *E. coli* through osmotic shock, as a result of Lf binding LPS [283]. With respect to Gram-positive bacteria such as *S. epidermidis*, the synergism between Lf and lysozyme was justified by Lf binding LTAs, which consequently reduced the negative surface charge of the bacterium [285]. The two proteins, when combined, were reported to induce a bactericidal effect against *S. pneumoniae* strains, potentially explained by the same mechanism [284]. In addition, the iron-binding state of Lf can influence the efficacy of its synergism with lysozyme, as the combination with apo-Lf was demonstrated to exert a higher antibacterial action [286]. Furthermore, studies suggest that Lf potentiated the anti-biofilm action of this enzyme. Thus, the combination of Lf and 8 and 16 mg/mL of lysozyme was effective in reducing the number of biofilm-embedded viable bacteria (0.46 and 0.59 log_10_ bacteria/mL) as opposed to lysozyme alone (–0.41 and –0.31 log_10_ bacteria/mL) [269]. Given their complementary mechanisms of action, Lf and lysozyme have been incorporated into emerging drug delivery systems such as liposomes and poloxamer 407 to optimize their combined effects against biofilm-forming cariogenic bacteria. For example, the Lf (0.001 g/mL)/lysozyme (0.002 g/mL) and poloxamer 407 formulation had the most notable effect on *S. mutans* and *Streptococcus sobrinus* biofilms [287]. Collectively, these findings support the potential use of the Lf-lysozyme mixture, as well as novel platforms incorporating these agents, for modulating biofilm formation in pathogenic bacteria.

In recent years, probiotic strains, including LAB, and their postbiotics (e.g., CFS, EVs, bacteriocins), have been considered as a complementary or even substitute agent for antibiotics, given their benefits [171]. For instance, *Lb. casei* and *Lb. rhamnosus* CFS were proven to work synergistically with aminoglycoside antibiotics in mitigating the growth of *P. aeruginosa* strains. These CFS consisted of H_2_O_2_, lactic acid, and acetic acid [288]. Additionally, CFS of probiotic strains of *B. subtilis* and *Bacillus amyloliquefaciens* displayed synergy with polymyxin E against *Acinetobacter* spp. biofilms [289]. Furthermore, the combination of *Lb. plantarum* CFS and levofloxacin had a better restrictive effect on preformed *H. pylori* biofilms as opposed to the individual antibiotic treatment. *Lb. plantarum* CFS also had an additive action when coupled with levofloxacin, which reduced the protein and polysaccharide contents of *H. pylori* biofilms [191].

Further reports support the use of postbiotics such as bacteriocins as complementary agents to antibiotics. Thus, bacteriocins produced by probiotic strains of *E. faecium* worked synergistically with ciprofloxacin toward the inhibition of *L. monocytogenes* biofilms, while the same bacteriocins had a synergic restrictive effect together with vancomycin on biofilm formation of VRE [290]. Moreover, bacteriocins have been proven to shift the antibiotic resistance phenotype of biofilm-embedded bacteria. For example, *E. coli* is nisin- and warnerin-resistant; warnerin is a lantibiotic produced by a probiotic strain of *Staphylococcus warneri*. However, the combination of bacteriocins and polymyxin B sensitized *E. coli* biofilms to the action of both agents. After a 24 h treatment with either warnerin plus polymyxin B or nisin plus polymyxin B, the cell viability within mature biofilms of *E. coli* decreased by five- to ten-fold. In particular, for the warnerin plus polymyxin B mixture, the bacteriocin decreased the effective concentration of the antibiotic from 18–36 µM to 4.5–9 µM. This evidence demonstrates that lantibiotics could be a promising alternative for mitigating antibiotic use, as they are non-toxic to eukaryotic cells [291].

Extracellular vesicles (EVs), as postbiotic components, have been investigated as adjuvants or novel delivery platforms to antibiotics, with the potential of enhancing antimicrobial and anti-biofilm efficacy. For instance, LAB EVs (LEVs) might be adjuvants to colistin by reducing its effective dosage without inducing resistance. Upon addition of LEVs, the bactericidal action of colistin was increased by four to eight-fold against *A. baumannii* strains. The LEVs/colistin combined use inhibited biofilm formation by 60–89%, whereas colistin alone induced a biofilm reduction of 36–48%, indicating that LEVs also enhanced the biofilm-restrictive activity of colistin [292]. Furthermore, LEVs have been used as drug delivery platforms for doxorubicin against *Staphylococcus* strains. Encapsulating doxorubicin into LEVs resulted in a more prolonged release, with values reaching 81.04% at pH 8.0 after 72 h, compared with the antibiotic alone, which, after 12 and 72 h, reached 86.81% and 95.29%, respectively. Additionally, LEV-incorporated doxorubicin had a six-fold higher activity than the antibiotic itself [293].

Based on the concept of synergistic antimicrobial strategies, recent studies have explored the co-treatment of probiotic-derived CFS with nanocarrier systems. In this context, chitosan nanoparticles (CsNPs) have been used to encapsulate CFS from *Lb. acidophilus* and *Bifidobacterium bifidum*, increasing their biological activities. Therefore, the novel co-treatment has been tested for its bactericidal action against MDR *Vibrio cholerae* isolates. The combined administration of both CFS–CsNPs revealed a synergistic bactericidal effect (MIC = 2.5 mg/mL), significantly lower compared with CFSa–CsNPs (25 mg/mL) and CFSb–CsNPs (5 mg/mL). A similar phenomenon was observed concerning biofilm inhibitory concentrations, which exceeded 200 mg/mL for the CFS-CsNPs alone, compared with the concomitant treatment (10 mg/mL). Additionally, the combination of CFSa–CsNPs and CFSb–CsNPs decreased bacterial adhesion to 11.19% and also inhibited internalization into Caco-2 cells [294]. In addition, within the development of novel postbiotic-based platforms, the encapsulation of bacteriocins into nanoparticle systems has emerged as a promising therapy against foodborne pathogens, as nanoparticle incorporation may potentiate activity and reduce the bactericidal concentrations required. The bacteriocin synthesized by *Lb. casei* showed synergism with iron oxide NPs against *E. coli* and *S. aureus*, and the combination induced cell membrane damage and pore formation in these pathogens [295].

The combination of postbiotics or probiotic metabolites with sterilization methods such as US or PDT has been explored as a novel strategy for eradicating MDR biofilms. The disruption of the cell membrane and the extracellular release of ATP were noted in the combination of either 0.5% or 1% of phenyllactic acid and US, which also displayed a synergic action by reducing exopolysaccharide contents in *S. aureus* biofilms [296]. The association between lactic acid and US rendered the best results in removing *E. coli* biofilms developed on lettuce leaves [297]. The synergism between methylene blue-mediated PDT and *Lb. acidophilus* CFS was evaluated as a new approach to reduce the incidence of MDR BAIs. The combination of PDT and LAB CFS induced membrane alterations and reduced viability in MRSA. Furthermore, the dual treatment also disrupted preformed biofilms and inhibited adherence compared with PDT and CFS alone [298].

The association between bacteriocins and biofilm-degrading enzymes has been evaluated as a new combined treatment targeted toward *L. monocytogenes* infections. The co-administration of the biofilm-degrading enzyme and thermophilin 110 dissociated mature biofilms and induced stress responses, illustrated by an altered cell morphology compared with each agent alone [200].

Natural bioactive compounds derived from plants have non-toxic effects. Plants belonging to the *Brassicaceae* family consist of phenolic and glucosinolate compounds with antimicrobial and antioxidant activities. Thus, the synergism between *Raphanus sativus* var. *longipinnatus* plant extracts and proteinase K has been proposed as a strategy for eradicating *E. coli* biofilms. When combined, the MBIC of *R. sativus* var. *longipinnatus* was reduced from 4 to 1 mg/mL, while the MBIC of proteinase K decreased from 1000 to 100 µg/mL, further highlighting their complementary interactions. The addition of 25 µg/mL proteinase K also improved the effect of *R. sativus* var. *longipinnatus* 2 mg/mL against biofilm formation of *E. coli* on a stainless-steel substrate, inducing a 2.68 log viability reduction [299].

## 5. Current Challenges, Perspectives, and Future Research Directions

Despite major advances in non-conventional anti-biofilm approaches, translation into routine clinical and industrial implementation remains limited by shared constraints: biofilm heterogeneity, adaptive resistance pathways, delivery barriers, safety/tolerability issues, and regulatory complexity. Importantly, biofilm communities deploy layered protective mechanisms that often blunt single-agent or single-modality interventions, reinforcing the need for integrative, multi-target designs [4,5,24,25,58]. This section synthesizes cross-cutting challenges, resistance mechanisms, and future directions for the previously presented anti-biofilm strategies, focusing on convergent bottlenecks and realistic innovation pathways. Across these strategy classes, recurring limitations highlight a shared principle: durable anti-biofilm control is most likely to emerge from rationally designed, multimodal interventions rather than standalone agents. To support this comparative perspective, Table 7 summarizes the translational maturity, strengths, limitations, clinical opportunities, implementation barriers, and future promise of the major anti-biofilm strategies discussed in this review.

Matrix-degrading enzymes represent a foundational anti-biofilm strategy by directly targeting the extracellular polymeric scaffold that confers mechanical stability and tolerance. However, their clinical implementation is constrained by context-dependent efficacy, which varies with biofilm composition, bacterial strain, growth conditions, enzyme concentration, and exposure time, while large-scale production, formulation stability, penetration into dense EPS, and cost remain significant barriers [4,66]. These limitations are particularly pronounced in mature biofilms, where restricted diffusion and limited substrate accessibility substantially reduce enzymatic activity. An additional challenge stems from the predominance of multispecies biofilms in clinical settings, where interspecies interactions can confer collective protection. In such communities, alterations in matrix composition or spatial organization by one population may shield partner strains from EPS-degrading enzymes, resulting in reduced or variable efficacy [66].

A critical and often underappreciated limitation is that enzymatic disruption of the EPS matrix can induce active biofilm dispersal rather than bacterial killing, releasing viable cells that retain colonization potential, thus creating a transient window of increased dissemination risk [25]. Consequently, matrix-degrading enzymes must be used in combination with other bactericidal agents to prevent dissemination and secondary infection. Multi-enzyme cocktails repeatedly outperform single-enzyme approaches, because they target distinct matrix components and achieve deeper and more sustained biofilm disruption across diverse bacterial species and substrates [4,5,25,67]. However, enzyme-antibiotic compatibility requires careful evaluation, as certain antibiotics may negatively affect enzyme stability or limit synergistic efficacy [70]. Collectively, these observations highlight that matrix degradation is most effective as part of rationally designed multi-target combinations, rather than empirical co-administration.

Future research directions focus on tailoring enzyme combinations to dominant matrix compositions, engineering enzymes with improved stability and substrate range, and developing advanced delivery platforms. Biocompatible polymers and nanocarrier-based systems (including polymeric nanoparticles, hydrogels, and enzyme immobilization strategies) have shown promise in improving penetration, preserving catalytic activity, and enabling sustained matrix disruption. The immobilization of enzymes such as alginate lyase, DNase I, and dispersin B within nanomaterials represents a promising approach for targeted and durable anti-biofilm intervention [25,66,67].

QSIs and QQ approaches represent a conceptually attractive anti-biofilm strategy by targeting bacterial communication and virulence regulation rather than viability. However, their translation faces important biological and practical limitations, as QS systems are highly species-specific and often hierarchically organized, with redundancy across signaling circuits, which limits the breadth and predictability of QS-targeting agents in complex or polymicrobial biofilms [26,28]. Moreover, QS inhibition is generally more effective at preventing biofilm formation than eradicating mature biofilms, which may rely less on active QS signaling [26].

Resistance to QS-targeting strategies does not typically arise through classical bactericidal selection pressures, but rather through adaptive regulatory rewiring or ecological buffering within multispecies communities. In addition, some QS inhibitors exhibit cytotoxicity, limited chemical stability, or uncertain long-term environmental effects, complicating their clinical and ecological deployment [26]. These constraints underscore that QS inhibition alone is therefore unlikely to achieve durable biofilm control in advanced infections.

Accordingly, QS-targeting agents have shown their greatest promise as adjuvant modulators. Synergistic effects have been reported when QS inhibition is combined with antibiotics, matrix-degrading enzymes, antimicrobial peptides, or advanced delivery systems that synchronize communication disruption with matrix penetration and bacterial killing [4,28,30]. For example, nanoparticle-based co-delivery platforms incorporating QS inhibitors and antibiotics improve biofilm penetration while reducing the antibiotic dose required for effective bacterial clearance [30]. Photoswitchable modulation of QS has emerged as an experimental strategy for spatiotemporal control of bacterial collective behavior using light as a non-invasive trigger. In these systems, light-responsive moieties, such as azobenzene units, are incorporated into autoinducer scaffolds, enabling reversible activation or silencing of QS signaling through photoisomerization. This approach decouples bacterial group behavior from fixed chemical inhibition and allows light-dependent control of Las-regulated virulence and biofilm-associated phenotypes in *P. aeruginosa* [28].

As a cross-cutting anti-persister direction, “wake-and-kill” strategies aim to transiently reactivate dormant subpopulations through controlled metabolic stimulation (using mannitol, glucose, or organic acids), thereby restoring susceptibility to bactericidal agents. Such approaches have significantly enhanced antibiotic efficacy against mature biofilms of *P. aeruginosa*, *E. coli*, *L. monocytogenes*, and *S. enterica* [95]. While careful temporal control is needed to prevent premature dispersal and increase dissemination risk, this strategy remains particularly attractive for industrial and device-associated biofilm control.

Future research should therefore focus on strategic optimization rather than standalone QSI development, including the synthesis of derivatives, targeted modification of existing QSIs, discovery of new QS inhibitors from natural products, repurposing of clinically approved drugs as QS-modulating agents [26], and programmable approaches that enable spatiotemporal control of QS activity [28]. Within the broader anti-biofilm framework, QS-targeting strategies are best positioned as modulatory tools that weaken biofilm coordination and virulence, thereby amplifying the effectiveness of physical, enzymatic, and antimicrobial interventions rather than replacing them.

Phage-based therapies and phage-derived antimicrobial molecules represent a biologically precise anti-biofilm strategy; however, their clinical translation is constrained by complex resistance mechanisms and practical limitations. These include EPS-mediated shielding, receptor decoys, receptor modification/concealment, intracellular exclusion, abortive defense systems, outer membrane vesicle (OMV) release, superinfection exclusion (Sie), retron-mediated defense, toxin–antitoxin systems, restriction–modification (R-M) systems, BacteRiophage EXclusion (BREX), abortive infection (Abi) pathways, and CRISPR–Cas immunity [29,30,68]. These multilayered defenses are particularly effective in mature biofilms, underscoring the need for careful phage selection and deployment.

In addition, the inadvertent use of filamentous or temperate phages can paradoxically enhance biofilm stability and virulence rather than disrupt it, by promoting liquid-crystalline structures within the matrix through interactions between phage particles, host cells, and microbial polymers [29]. Beyond biological resistance, additional barriers include narrow host range, incomplete pharmacokinetic and pharmacodynamic characterization, immunogenicity, regulatory and ethical constraints (particularly for genetically modified phages), as well as challenges related to large-scale production, formulation, storage, and targeted delivery [24,58].

Consistent with these limitations, phage therapy is most effective when implemented as part of combination strategies rather than as a standalone intervention. Phage-antibiotic synergy (PAS) has been repeatedly demonstrated, whereby phage-mediated matrix disruption enhances antibiotic penetration, while sublethal antibiotic exposure increases phage infectivity and replication, resulting in improved killing of biofilm-associated and MDR bacteria [5,58]. Similarly, phage cocktails comprising multiple lytic phages with complementary host ranges reduce resistance emergence and improve biofilm eradication compared with monophage treatments [29,68].

The field is increasingly shifting toward phage engineering and delivery optimization as prerequisites for clinical translation. Advances in synthetic biology have enabled phages engineered to express matrix-degrading enzymes, QQ molecules, anti-CRISPR or anti-R-M proteins, thereby enhancing penetration, replication efficiency, and resistance evasion within biofilms [29,30]. In parallel, broad-host-range phage discovery and nanotechnology-assisted formulations may improve stability and controlled release, positioning phage-based approaches as a powerful yet context-dependent component of next-generation anti-biofilm strategies [4].

CRISPR-Cas and nanotechnology-assisted delivery represent emerging programmable directions for anti-biofilm intervention. CRISPR-Cas-based antimicrobials have the potential to selectively eliminate resistance determinants, restore antibiotic susceptibility, or target biofilm-regulatory pathways. For example, CRISPR-Cas systems have been explored for targeting resistance genes such as *mecA* in MRSA, while CRISPR interference has been used to silence QS-related genes in *P. aeruginosa*, reducing matrix formation. However, delivery efficiency, off-target effects, and possible immune responses remain major translational barriers, suggesting that CRISPR-Cas approaches are best positioned within combination or nanotechnology-assisted delivery platforms [31].

AMPs represent a versatile anti-biofilm strategy with rapid, multi-target modes of action; however, their clinical translation remains constrained by resistance, stability, safety, and delivery limitations. Bacteria may respond to AMP susceptibility through envelope structure remodeling, peptide degradation, efflux activation, membrane shielding, or surface modifications, supporting the need for careful resistance monitoring [58,69,300]. In parallel, cytotoxicity or off-target effects at higher concentrations, proteolytic instability, short half-life, high production costs, potential immunogenicity, and limited diffusion through dense EPS matrices continue to restrict their broader application, particularly in advanced BAIs [3,5,69,301].

Accordingly, AMPs show their greatest therapeutic potential when implemented as part of rational combination or delivery-assisted strategies rather than as single-agent interventions. Synergistic effects have been reported when AMPs are combined with antibiotics, matrix-degrading enzymes, QS inhibitors, chelating agents, bacteriocins, or nanocarrier-based delivery systems, enabling dose reduction, improved biofilm penetration, and enhanced bactericidal activity [4,5,22,58]. Such combinations can suppress multiple biofilm-supporting pathways simultaneously and mitigate resistance emergence.

Future research directions should focus on rational AMP design and formulation rather than de novo peptide alone, including peptide engineering for improved stability and selectivity, chemical modification to reduce cytotoxicity, localized delivery through nanocarriers, biomaterials, or surface coatings, development of AMP-antibiotic or AMP-enzyme hybrids, and the use of recombinant DNA technology [5,58,69,301]. Within the broader anti-biofilm framework, AMPs are best positioned as adaptable components of multimodal therapies that integrate direct antimicrobial activity with anti-virulence and immune-modulatory effects.

Despite their proven inhibitory effects, Gram-positive and Gram-negative pathogens have acquired multiple resistance mechanisms or strategies to block the antimicrobial and anti-biofilm activities of Lf and Lf peptides [120]. For instance, several Gram-negative enteric bacteria can display phenotypic resistance against Lf, justified by the potential shielding of membrane porin receptors by *O*-antigenic chains of LPS [302]. Gram-positive bacteria such as *S. pneumoniae* and *S. aureus* can bind the active sites of apo-hLf and LFcin through surface proteins and adhesins, thus blocking their bactericidal action [300,303]. Additionally, bacteria can hijack Lf-bound iron by synthesis of high-affinity siderophores, by bacterial reductases which convert Fe^3+^ to Fe^2+^, and via iron acquisition by Lf receptors [304]. For example, *Neisseria* spp. possesses Lf surface receptors, which are responsible for iron piracy from hLf and can also counteract the attachment of LFcin [300,304,305,306,307,308]. In this context, the combined use of Lf or Lf-derived AMPs with other natural compounds, antibiotics, or antimicrobial strategies might provide a promising tool for overcoming bacterial evasion of Lf activity.

Postbiotics synthesized by probiotic strains have attracted interest for the treatment of BAIs. However, in the absence of efficient carriers targeting biofilm-embedded bacteria, postbiotics might not be ideal substitutes for classical anti-biofilm agents, but rather complementary strategies [182]. In addition, the synthesis of postbiotics is not constant, since their composition and efficacies might vary between batches, which further supports their use as adjuvants rather than independent standardized drugs [182]. Furthermore, pathogens have developed strategies to evade the action of several postbiotics, such as bacteriocins, including the alteration of cell wall composition and anionic charges, as well as membrane permeability and fluidity. Though bacteriocins are considered non-toxic, in the framework of postbiotics, resistance to bacteriocins should be taken into consideration and further researched, together with exploring new approaches to limit resistance and allow the safe use of bacteriocins [309].

Despite the growing interest in natural bioactive compounds as anti-biofilm agents, several important limitations remain. Most current data are derived from in vitro studies, which do not fully replicate the complexity of biofilms. Consequently, these models are insufficient to accurately predict the efficacy under *in vivo* conditions, particularly in the context of multispecies biofilms that better reflect real-world citadels. The limited number of in vivo studies restricts the understanding of the pharmacokinetics, metabolism, and long-term biocompatibility of phytochemicals.

Another major limitation is the inconsistent efficacy reported for natural compounds. Variability in plant sources, extraction methods, chemical composition, and experimental protocols leads to a lack of standardization, making it difficult to compare results across studies. In addition, some phytochemicals may present allergenic or cytotoxic effects, raising concerns regarding their safety and clinical applicability [227,310,311].

Furthermore, several natural compounds have been reported to display poor bioavailability, rapid degradation, and low water solubility, which significantly limit their anti-biofilm effectiveness. Certain challenges have been reported in polymicrobial biofilms, where increased microbial virulence, metabolic cooperation, and enhanced tolerance mechanisms reduce susceptibility compared with monospecies biofilms. As observed by Gobin et al. (2022) [230], combinations that are effective against single-species biofilms often require substantially higher concentrations to achieve comparable eradication in multispecies biofilms.

Overall, these limitations emphasize the urgent need for standardized methodologies, advanced in vivo and multispecies biofilm models, and improved delivery strategies. Approaches such as combination therapies with antibiotics or other natural agents show promise in enhancing complementary or even synergistic anti-biofilm effects but require systematic and standardized investigation before clinical application [267,312].

More recently, micro- and nano-motor-based technologies have emerged as mechanical stress-driven anti-biofilm strategies, including magnetically driven microbots, light-activated molecular motors, and ultrasonic micromotors capable of penetrating and disrupting biofilm matrices through localized mechanical action. Their autonomous motion and enhanced biofilm penetration enable direct targeting of biofilm mechanical stability and support integration with antimicrobial or photonic therapies. Although translation remains limited by regulatory, biosafety, and scalability challenges, these systems represent promising complements to chemical and biological anti-biofilm approaches, particularly for difficult-to-remove biofilms in medical and industrial contexts [4].

Collectively, these observations support a paradigm shift from agent-centric anti-biofilm strategies toward context-aware multimodal strategies with improved delivery systems, adapted to biofilm structure, composition, and physiological state.

## 6. Conclusions

Biofilm-associated infections remain a major public health concern due to increased antimicrobial tolerance, persistence, and the development of phenotypic and genetic resilience. The evidence summarized in this review highlights the potential of combinatorial strategies involving natural bioactive compounds (lactoferrin, probiotics, postbiotics, and phytoconstituents) and/or standardized agents to enhance anti-biofilm efficacy through complementary mechanisms of action.

Distinguishing between biofilm prevention and eradication, mitigating risks such as dispersal without killing, and accounting for the evolutionary consequences of tolerance-driven survival mechanisms are essential for the translational success of any future therapy. Strategies that fail to integrate these considerations may inadvertently promote persistence or resistance development.

Despite the promising findings summarized in this paper, several limitations still hinder the clinical application of these multimodal strategies. For example, most of the presented research is limited to in vitro experimental models, while outcome measures and experimental conditions are heterogeneous across studies. Future research should focus on standardizing emerging agents such as postbiotics, conducting mechanistic investigations, and validating combined strategies using in vivo models.

Looking forward, progress in anti-biofilm therapy will depend less on the identification of new antimicrobial agents and more on the development of adaptive and combination-based strategies that address biofilm developmental stages and constrain evolutionary escape pathways. Embracing a systems-level approach to biofilm control is essential for addressing microbial tolerance and persistence, and for intercepting the evolutionary trajectories that link biofilm survival to antimicrobial resistance development.

## Figures and Tables

**Table 1:** Quorum-sensing signaling molecule classes and anti-QS/quorum-quenching agents.

QS Signaling Molecule Class/System	Anti-QS Molecules/QQ Enzymes Reported	QQ/QSI Mechanism of Action	Effect on Biofilm/Virulence	Sources
AHLs (N-acyl homoserine lactones; LuxI, LuxR, and HdtS systems in Gram-negative bacteria)	AiiA, fusaric acid, phenazine carboxylic acid, citrinin	AiiA degrades AHLs; fusaric acid, phenazine carboxylic acid, and citrinin interfere with AHL-mediated QS signaling.	Reduced AHL-dependent communication and QS-regulated virulence/biofilm behaviors in *V. fischeri* and *P. aeruginosa*.	[74,75,76,77]
AIPs (peptide autoinducers; *agr* system of *S. aureus* and Gram-positive bacteria)	Cyclodepsipeptides WS9326A, WS9326B, cochinmicin II/III, N-(pyridin-2-yl)-benzamides derivatives, AIP analogues	Peptide and small molecules antagonize AIP–receptor signaling and modify peptide–receptor interactions.	Reduced expression of Agr-dependent virulence factors (e.g., α-hemolysin, δ-toxin) and biofilm-related traits in *S. aureus*; altered competence and bacteriocin production in *Bacillus* spp. and *L. monocytogenes*.	[77,78,79]
AI-2 (universal signal for interspecies communication; LuxP/LuxQ system in *Vibrio* spp.; LsrB/Lsr system in Enterobacteriaceae; S-THMF-borate and R-THMF signals in *Vibrio harveyi*; KinD in *Bacillus subtilis* and PctA in *P. aeruginosa*)	Moracin M, cannabigerolic acid, piperine	Natural compounds target AI-2 signaling in *V. harveyi* by affecting AI-2–dependent pathways.	Reduced AI-2–mediated interspecies communication, biofilm development, and virulence-associated behaviors.	[77,80,81,82]
AI-3 (3,6-dimethylpyrazin-2(1H)-one; QseCB system; in enterohemorrhagic *E. coli* and Gram-negative bacteria)	Fructose furoic acid, isolimonic acid, esculetin	Target QseC-dependent QS signaling, decreasing toxicity, and global regulation of AI-3-controlled regulatory outputs.	Reduced toxicity and virulence in AI-3–QseC-regulated infections, including *E. coli*, *Salmonella* spp., and *V. parahaemolyticus* AHPND.	[77,83]
AQs (alkyl-quinolones; PQS system; in *P. aeruginosa*)	Quinazolinone disulfide analogues, clofoctol, paecilomycone	Inhibit PQS biosynthesis and regulatory circuits.	Reduced pyocyanin and other phenazine production; reduced biofilm formation and virulence; PQS pathway inhibition linked to biofilm inhibition.	[77,84]
DSF (diffusible signal factor; RpfC-RpfG system; in *Xanthomonas* spp.)	Zingerone derivatives	DSF analogues interfere with DSF signaling and perception.	Reduced virulence, biofilm architecture, and antibiotic resistance in *X. campestris*.	[77,85]
DKP (2,5-diketopiperazines; LuxR-related system; in *Shewanella baltica*)	Not reported	Not reported; DKPs are described as positive transcription regulators.	DKPs positively regulate transcription and spoilage-related traits; anti-QS effects not reported.	[77,86]
CAI-1 (cholera autoinducer-1: (S)-3-hydroxytridecan-4-one; CqsS system; in *Vibrio cholerae* and *Photobacteria* spp.)	Isonaamidine A, isonaamine D	Inhibit CAI-1 signal–receptor specificity and antagonize CAI-1-mediated QS signaling.	Modulate QS-dependent virulence in *Vibrio* spp.; specific anti-QS effects on biofilm not reported.	[77,87,88]
Emerging signaling molecules (indole, 3-OH-PAME, γ-butyrolactones, DARs, 2-AA)	Carnosol (for indole), ELP86, ELP96, ELP104, EstDL33 (for 3-OH-PAME)	Carnosol targets indole signaling and interferes with indole-mediated regulatory pathways; esterases degrade 3-OH-PAME and act as QQ agents; specific QQ/QSI mechanisms are not reported yet for other emerging signaling molecules.	Carnosol reduces biofilm formation, virulence, and stress tolerance in *L. monocytogenes*; 3-OH-PAME-degrading esterases modulate virulence factors and contribute to wilt prevention in *Ralstonia solanacearum*.	[77,89,90,91,92,93,94]

**Table 2:** Inhibitory effects of Lf and Lf-derived peptides on bacterial biofilm formation.

Bacterial Pathogen	Type of Lf Tested	Reported Effect	Outcome Measure	References
*P. aeruginosa*	bLf	Anti-biofilm effect	0.1–2 mg/mL	[32]
		Biofilm eradication	MBEC = 62.5 µg/mL	[33]
		72.35% biofilm formation inhibition	14 mg/mL	[34]
	Food supplement-derived Lf	Biofilm eradication	MBEC = 1250 µg/mL	[33]
	LFcinB	Biofilm eradication	MBEC = 128 µM	[153]
*E. coli*	bLf	Moderate biofilm inhibition	16 mg/mL	[34]
		Biofilm eradication	MBEC = 2000 µg/mL	[33]
	Food supplement-derived Lf	Biofilm eradication	MBEC = 2000 µg/mL	
*S. aureus*	bLf	41.47% biofilm formation inhibition	8 mg/mL	[34]
		Biofilm mass reduction	1 mg/mL, 10 mg/mL	[156]
		Biofilm eradication	MBEC = 2000 µg/mL	[33]
	bLf hydrolysate	71% biofilm reduction	2.5 mg/mL	[157]
	Food supplement-derived Lf	Biofilm eradication	MBEC = 2000 µg/mL	[33]
*E. faecium*	Synthetic LFcinB-derived peptides	90% biofilm formation inhibition	32 μg/mL	[143]
	bLf	Biofilm eradication	MBEC = 250 µg/mL	[33]
	Food supplement-derived Lf	Biofilm eradication	MBEC = 1250 µg/mL	
*P. gingivalis*	bLf	Biofilm formation inhibition	0.031 mg/mL, 0.5 mg/mL	[146]
		>80% biofilm formation inhibition	>0.625 µM	[147]
*P. intermedia*	hLf	Biofilm formation inhibition	>0.13 mg/mL	[146]
	bLf	Biofilm formation inhibition	>0.031 mg/mL	
	LFcinB	Biofilm formation inhibition	0.4 mg/mL	
*S. pneumoniae*	bLf	Biofilm eradication	40 µM	[130]
*S. agalactiae*	hLf (apo- and holo-)	Apo-hLf: 50% biofilm formation inhibition Holo-hLf: 42% biofilm formation inhibition	250 μg/mL (apo- and holo-hLf)	[158]
*Bacteroides* spp.	Recombinant hLf	Biofilm formation inhibition	12.5 µg/mL	[145]
*Listeria* spp.	bLf	~60% biofilm formation inhibition	6 mg/mL	[34]
*A. baumannii*	hLf	74% biofilm formation inhibition	125 μg/mL	[159]
	bLf	55% biofilm formation inhibition	125 μg/mL	
*S. enterica*	bLf	50% biofilm viability reduction	40 μM	[160]
	bLf-chimera	80% biofilm viability reduction	10 μM	
	bLFampin	70% biofilm viability reduction	10 μM	

**Table 5:** Combinations of natural bioactive compounds with anti-biofilm activity against clinically relevant pathogens.

Combination Treatment	Targeted Biofilm-Forming Microorganisms	Individual Compound Activity	Quantitative Anti-biofilm Effect	Potential Synergistic Mechanisms	Experimental/Clinical Relevance	References
Terpinen-4-ol + α-Terpineol (10:1)	*S. aureus* MRSA (CIP10742) *E. coli* (CIP53.126) *P. aeruginosa* (CIP82.118) Monospecies biofilms	Terpinen-4-ol: MIC = 0.31–2.50% +α-terpineol: MIC = 0.15–2.50%	FICI = 0.093–0.375 (Synergistic activity)	Membrane destabilization; Impairment of EPS production; Inhibition of motility and protease activity; QS interference.	In vitro activity against ESKAPE-associated pathogens	[31]
Gallic acid + Carvacrol (0.5–2.0 mg/mL and 0.5–5.0 mg/mL)	Mono-and dual-species mature biofilms of *S. aureus* and *P. aeruginosa* (2:1)	Gallic acid: MIC = 2.5 mg/mL Carvacrol: MIC = 0.128–2.0 mg/mL	3.7–5.4 log reduction (dual-species biofilms; (conc. 0.5–2.0 mg/mL)	Gallic acid exhibits anti-adherence activity, destabilizing the biofilm matrix, enhancing carvacrol diffusion and activity → membrane disruption, cellular leakage, and cell death.	In vitro wound biofilm model	[217]
Thymol + Carvacrol (1/4 × MIC)	Monospecific biofilms of *S. aureus* MSSA ATCC 6538	Thymol: MIC = 256–2048 µg/mL Carvacrol: MIC = 128–1024 µg/mL	FICI = 0.50 (Synergistic activity) ~74.6% biofilm removal	Dual Membrane disruption; Increased cell permeability; Lipid bilayer damage → biofilm integrity disruption.	In vitro treatment model for BAIs	[246]
α-Terpineol + Nerolidol (1:1)—1 mg/mL	Monospecific biofilms of *E. coli* 82 (isolated from HS)	α-Terpineol: mean MBEC = 1000 µg/mL Nerolidol: mean MBEC = 1000 µg/mL	MBEC = 7.8125 μg/mL	Membrane targeting effects via hydrophobic interactions → increased permeability and cell lysis—attributed to nerolidol. QS inhibition—associated with α-terpineol.	In vitro (Potential therapeutic approach for chronic wound management)	[26]
Carvacrol + Linalool	Polymicrobial *Gardnerella* spp. biofilms	Linalool: MIC = 1.25–5 µL/mL Carvacrol: MIC = 0.16–5 µL/mL	FICI = 0.50 (Synergistic activity) ~50% biomass reduction at sub-MIC concentrations (0.02–0.32 µL/mL)	Synergistic membrane disruption; Enhanced penetration; Destabilization of biofilm matrix and cellular integrity.	In vitro (potential topical application for treating bacterial vaginosis)	[222]

**Table 6:** Combinations of natural bioactive compounds and antibiotics with anti-biofilm activity against clinically relevant pathogens. Interactions, based on the FICI value, are classified as: FICI ≤ 0.5: synergy; 0.5 < FICI ≤ 1: additive; 1 < FICI ≤ 4: indifferent; FICI ≥ 4.0: antagonistic.

Combination of Compounds and Antibiotics	Targeted Microorganisms	Combined Effect	Potential Mechanism	Model and Clinical Application	References
LFcinB (NS ^1^) + erythromycin (NS)	*E. coli*	FICI = 0.3 (Synergism)	Enhancement of the bactericidal action of erythromycin.	In vitro (combined treatment for the clinical management of bacterial infections)	[139]
Lf (NS) + vancomycin (4–64 µg/mL)	*S. epidermidis*	Reduction of the viable bacteria count by 3.57 log_10_ bacteria/mL compared with vancomycin individually (1.85 log_10_ bacteria/mL);	Enhancement of the bactericidal action of vancomycin.	In vitro (Lf as an adjuvant in eradicating *S. epidermitis* biofilms)	[269]
rhLf (900 mg/L) + rifampicin (NS)	*Burkholderia cepacia* complex	Decrease in the BIC of rifampicin (128–256 mg/L) in the presence of rhLf (16 mg/L)	Enhancement of biofilm susceptibility to rifampicin.	In vitro (therapeutic alternative for the clinical management of cystic fibrosis)	[270]
LFcinB (NS) + ciprofloxacin (NS)	*P. aeruginosa*	FICI = 0.5 (Synergism)	Membrane permeabilization; ciprofloxacin favors the interaction of LFcinB with the outer membrane.	In vitro (antibacterial therapy for contact lens-associated corneal infections)	[264]
LFcinB (6.25–25 µg/mL) + erythromycin (NS)	*P. aeruginosa*	Decrease in the MIC of erythromycin (128 µg/mL) in the presence of LFcinB (1 µg/mL)	Overexpression of MDR efflux pumps; loss of porins.	In vitro	[271]
cLf (camel Lf) (1.95 µg/mL–2 mg/mL) + vancomycin (0.06–30 µg/mL) cLf (camel Lf) (1.95 µg/mL–2 mg/mL) + oxacillin (0.25–100 µg/mL)	MRSA	FICI = 0.37 (Synergism) FICI = 0.5 (Synergism)	Enhancement of cell membrane disruption.	In vitro (the use of cLf individually or combined with antibiotics against MRSA)	[43]
LFchimera (0.125–0.25 μM) + doxycycline (1.4–2.8 μM)	*A. actinomycetemcomitans*	FICI = 0.25–0.38 (Synergism) Reduction in bacterial adhesion by 87%, mitigation of biofilm mass and thickness	Inhibition of bacterial adhesion; membrane permeabilization	In vitro (adjuvant in the treatment of periodontal disease)	[272]
Carvacrol + chloramphenicol; (1/4 × MIC)	*S. aureus* MSSA ATCC 6538	FICI = 0.50 (Synergism) 79.3% biofilm reduction	Membrane permeabilization facilitates antibiotic diffusion and subsequent inhibition of protein or DNA synthesis; preformed biofilm removal.	In vitro (targeted treatments for biofilm-related infections)	[267]
Thymol + chloramphenicol (1/4 × MIC)		FICI = 0.50 (Synergism) 78.3% biofilm reduction			
α-Terpineol (0.15 μg/mL) + Gentamicin (0.25 μg/mL)	*P. aeruginosa* PAO1	FICI = 0.1875 (Synergism) Elimination of preformed *Pseudomonas* biofilms (young: 68.5%; mature: 64.8%; old: 67.2%) is superior to individual treatments; antibiotic alone → ineffective	Eradication of preformed biofilms; gentamicin enhances the QQ potential of α-terpineol, interfering with AHL production, and inhibiting virulence factors, while exerting anti-fouling activity through suppression of EPS and protein biosynthesis.	In vitro (model for treating healthcare -associated infections); in vivo (73% survival in *Caenorhabditis elegans* infected with *P. aeruginosa*)	[44]
Cefixime (5.33 µg/mL) + Thymol (32 µg/mL)	*S. aureus* ATCC 1026 *S. epidermidis* ATCC 35984 *S. aureus*, *S. epidermidis*, *and S. saprophyticus* isolated from urine samples	FICI ≤ 0.50 (Synergism) for both biofilm inhibition and eradication;	Thymol possesses the ability to disrupt the cell membrane and inhibit QS, promoting antibiotic penetration.	In vitro (potential therapeutic application as antimicrobial adjuvants for UTI-related infections)	[273]
Cefazolin (1.16 µg/mL) + Thymol (21.33 µg/mL);		FICI = 0.30 (Synergism) (*S. saprophyticus*)—for biofilm inhibition; FICI = 0.44 (Synergism) (*S. epidermidis*)—for biofilm inhibition; FICI = 0.31 (Synergism) (*S. epidermidis*)—for biofilm eradication;			
Curcumin + Colistin	*A. baumannii* ATCC 17987 31 strains of *A. baumannii* MDR (isolated from blood, urine, sputum, wound)	FICI = 0.25–1.12 (Synergism) 13.3–32.1% biofilm inhibition (0.0078 × 1024 μg/mL), 9.1% biofilm eradication (0.08 μg/mL/2048 μg/mL)	Potential enhanced inhibition through intracellular penetration of curcumin due to colistin-induced outer membrane disruption.	In vitro (adjunctive anti-biofilm strategy against BAIs caused by MDR *A. baumannii*)	[274]
Geraniol + Colistin		FICI = 0.12–1.03 (Synergism) 12.05–41.03% biofilm inhibition (0.004 × 64 μg/mL) 20.9% biofilm eradication (0.0312 μg/mL/256 μg/mL)	Potential membrane permeabilization and efflux inhibition by Geraniol, enhancing colistin uptake and intracellular accumulation.		
Linalool + Colistin		FICI = 0.14–1.06 (Synergism) 11.03–35.8% biofilm inhibition (0.0078 × 512 μg/mL) 22.5% biofilm eradication (0.0312 μg/mL/1024 μg/mL)	Potential membrane interaction and modulation of adhesion and QS systems by Linalool, facilitating colistin penetration and enhancing biofilm efficacy.		
Cranberry proanthocyanidin (100 µg/mL) + sulfamethoxazole (512 µg/mL)	*E. coli* CFT073	FICI ≤ 0.50 (Synergism) Significant reduction of biofilm biomass	Increased cell membrane permeability, inhibition of efflux pumps; Cranberry proanthocyanidin impairment of dormant antibiotic-exposed cells.	In vitro (potential application in device and tissue-associated infections); In vivo (insect models—*Drosophila melanogaster*, increased survival)	[275]
Small Molecular Adjuvants (SMA) (obtained by chemical synthesis) (16 mg/mL) + Fusidic acid (4 mg/mL)	Multispecies biofilms of *A. baumannii*, *S. aureus* MRSA, *S. aureus*, and *P. aeruginosa*);	FICI = 0.08–0.13 (Synergism) Significant reduction of biofilm biomass (~70–75%)	Membrane permeabilization and efflux pumps inhibition leading to increase concentration of antibiotics within biofilms and destabilization of the EPS matrix.	In vitro (potential treatment of chronic BAIs caused by MDR pathogens); In vivo (antibacterial activity in a mouse skin infection model of *A. baumannii*)	[276]
Quercetin (125 µg/mL) + Amikacin (NS)	*P. aeruginosa* (YU-V10, YU-V11, YU-V15 and YU-V28)—isolated from catheter-associated UTI *P. aeruginosa* PAO1	FICI = 0.25–0.5 (Synergism) Inhibition of biofilm formation by reducing 90% of the total biomass	The combination potentially facilitates biofilm matrix penetration and intracellular antibiotic accumulation, leading to cell death. Quercetin inhibits QS systems, rhamnolipid synthesis, swarming motility, and virulence factor expression.	In vitro (potential applicability for catheter-associated UTI management)	[277]

^1^ NS = Not Specified.

**Table 7:** Translational comparison of major anti-biofilm strategies. The table highlights strategy maturity, strengths, limitations, clinical opportunities, implementation barriers, and future promise, and supports a shift from standalone anti-biofilm agents and strategies toward rational, localized, multimodal interventions adapted to biofilm maturity, infection site, microbial composition, matrix structure, and delivery feasibility.

Maturity of Strategy	Translational Strengths	Main Limitations	Clinical Opportunity	Clinical Threats/Implementation Barriers	Future Translational Promise	References
Antibiotics/conventional chemotherapeutics						
Clinically established.	Direct antimicrobial activity; Broad availability; Established dosing frameworks; Compatible with combination therapy.	Reduced efficacy in mature biofilms; Poor penetration; Persister tolerance; Toxicity limits dose escalation; Resistance selection.	BAIs requiring antimicrobial therapy; Core therapy in combination strategies; Useful after improved biofilm accessibility.	Therapeutic failure in mature biofilms; Mismatch between in vitro susceptibility and in vivo effects; AMR selection; Ecological residue concerns.	Well established but limited: clinically indispensable, but insufficient alone.	[3,5,15,16,17,22,48,54,56]
Physical approaches (mechanical debridement, US, PDT, PTT)						
Clinically used, mainly as an adjunct strategy.	Rapid biomass reduction; Local effect; Improved access; Low target-specific resistance pressure.	Incomplete eradication; Viable-cell dissemination; Parameter sensitivity; Limited penetration for PDT; Tissue damage risk.	Chronic wounds, dental/oral biofilms, device-associated biofilms, accessible localized infections; Adjunct to antibiotics, antiseptics, or delivery systems; Useful in device decontamination.	Lack of standardized protocols; Recurrence after incomplete removal; Local toxicity; limited use in inaccessible or deep layers of biofilms.	High, near-term as adjunct strategy: clinically relevant when integrated with antimicrobial strategies.	[4,28,49,50,51,52,53,60,61,64]
Matrix-degrading enzymes						
Mainly preclinical/in vitro.	EPS weakening; Improved penetration; Multi-enzyme cocktails; Compatible with other agents.	Limited standalone killing; Substrate specificity; Reduced access in dense or multispecies biofilms; Instability, formulation, cost, delivery, and compatibility issues.	Wound, device-associated, and surface-associated biofilms; Adjunct to antibiotics, phages, AMPs, antiseptics, or immune clearance; Topical formulations and coated surfaces.	Dispersal of viable cells; Variable efficacy in multispecies biofilms; Enzyme-antibiotic incompatibility; Regulatory complexity for enzyme cocktails.	High conditional: strong adjunctive potential if paired with bactericidal strategies and targeted delivery.	[4,5,25,66,67,70]
QS/QQ and anti-virulence strategies						
Mainly preclinical/in vitro.	Virulence attenuation and reduced coordination; Low direct bactericidal pressure; Good adjuvant compatibility.	More effective for prevention than eradication; Species-specificity; Redundant QS networks; Limited use in polymicrobial biofilms; Stability and cytotoxicity concerns.	Biofilm prevention and maturation control; Adjunct to antibiotics, enzymes, AMPs, or delivery systems; Anti-adhesion coatings.	Regulatory uncertainty for anti-virulence endpoints; Adaptive regulatory pathways; Ecological buffering in multispecies biofilms; Uncertain long-term environmental effects.	Moderate to high conditional: promising as a modulatory adjunct, not as a standalone eradication strategy.	[4,5,22,26,28,30,95]
Phages and phage-derived enzymes						
Preclinical/early translational.	High bacterial specificity and lytic activity; Depolymerases and endolysins activity; Cocktails broaden host range.	Narrow host range; phage resistance; Immunogenicity; Formulation, production, storage, and delivery challenges.	Personalized or localized treatment of wound, device-associated, and refractory bacterial biofilms; Adjunct to antibiotics or matrix-disrupting agents; Coatings and surface decontamination.	Regulatory and ethical complexity; Variable host–phage interactions; Risk of inappropriate phage selection; Potential horizontal gene transfer with temperate phages.	High conditional: strong translational potential, but dependent on phage selection, delivery, and resistance management.	[3,4,5,27,29,30,31,58,68]
AMPs and bacteriocins						
Mainly preclinical/in vitro.	Fast multi-target activity; Potential anti-persister effects; Synergy with other strategies and delivery systems.	Cytotoxicity at higher concentrations; Proteolytic instability; Short half-life; Production cost; Limited diffusion through dense EPS; Possible resistance/cross-resistance.	Localized treatment of wound, oral, skin, and device-associated biofilms; Adjunct to antibiotics, enzymes, QSIs, or nanocarriers; Coatings and biomaterials.	Safety and selectivity concerns; Formulation instability; Scalability; Resistance or cross-resistance monitoring; Limited systemic applicability.	High conditional for local and delivery-assisted use; less promising as a standalone systemic therapy.	[3,4,5,22,24,58,69,98,99,300]
Plant-derived bioactive compounds, postbiotics, lactoferrin, and lactoferrin-derived peptides						
Mainly preclinical/in vitro.	Multi-target activity; Anti-adhesion and immune modulation; Adjuvant potential; Consumer acceptability; Good preventive profile.	Variable composition; Variable efficacy; Poor solubility or bioavailability; Batch variability; Limited in vivo validation; Standardization challenges.	Preventive, topical, oral/dental, wound, skin, food-related, and device-surface applications; Adjunct to antibiotics, LAB-derived fractions, PDT/US, enzymes, or nanocarriers; Functional formulations.	Regulatory classification difficulties; Inconsistent methodologies; Limited pharmacokinetic and biocompatibility data; Reduced predictability in multispecies biofilms.	Moderate to high conditional: strongest as preventive, topical, or adjuvant strategies; weaker as standardized standalone therapeutics.	[24,33,37,39,105,121,182,183,188]
Advanced delivery and programmable platforms (nanocarriers, enzyme immobilization, engineered phages, photoswitchable QS tools, wake-and-kill strategies, micro-/nanomotors)						
Future translation.	Improve delivery; Controlled release; Enhanced penetration; Spatial targeting; Multimodal integration.	Technical complexity; Off-target effects; Biosafety concerns; Scalability; uncertain Biodistribution; Limited in vivo validation.	Localized biofilm therapy, wound dressings, catheter/device difficult-to-remove biofilms; Delivery layer for enzymes, phages, AMPs, QSIs, CRISPR-Cas tools, or metabolic stimulants; Smart coatings and responsive biomaterials.	Regulatory complexity; Environmental and biosafety concerns; Standardization and cost barriers.	High but early-stage conditional: strongest as an enabling layer for multimodal therapy, not as an independent strategy.	[4,25,28,29,30,31,66,67,68,95]

## Data Availability

The original contributions presented in this study are included in the article. Further inquiries can be directed to the corresponding author.
